# Vascular Cells Proteome Associated with Bradykinin and Leptin Inflammation and Oxidative Stress Signals

**DOI:** 10.3390/antiox9121251

**Published:** 2020-12-09

**Authors:** Moustafa Al Hariri, Miran A. Jaffa, Richard Saoud, Jingfu Zhao, Rui Zhu, Aneese A. Jaffa, Ghewa A. El-Achkar, Mayssam Moussa, Firas Kobeissy, Anwarul Hassan, Fuad N. Ziyadeh, Yehia Mechref, Ayad A. Jaffa

**Affiliations:** 1 Department of Biochemistry and Molecular Genetics, Faculty of Medicine, American University of Beirut, Beirut 11-0236, Lebanon; ma147@aub.edu.lb (M.A.H.); rss48@aub.edu.lb (R.S.); ga77@aub.edu.lb (G.A.E.-A.); mm314@aub.edu.lb (M.M.); fk02@aub.edu.lb (F.K.); fz05@aub.edu.lb (F.N.Z.); 2 Epidemiology and Population Health Department, Faculty of Health Sciences, American University of Beirut, Beirut 11-0236, Lebanon; ms148@aub.edu.lb; 3 Department of Chemistry and Biochemistry, Texas Tech University, Lubbock, TX 79409, USA; jingfu.zhao@ttu.edu (J.Z.); rui.zhu@abbvie.com (R.Z.); 4 Faculty of Arts and Sciences, American University of Beirut, Beirut 11-0236, Lebanon; aaj39@mail.aub.edu; 5 Department of Mechanical and Industrial Engineering, Qatar University, Doha 2713, Qatar; ahasan@qu.edu.qa

**Keywords:** bradykinin, inflammation, kallikrein kinin system, leptin, mitochondrial dysfunction, oxidative stress, proteomics, systems biology

## Abstract

Among the primary contributors to cardiovascular diseases are inflammation and oxidative imbalance within the vessel walls as well as the fibrosis of rat aortic smooth muscle cell (RASMC). Bradykinin (BK) and leptin are inflammatory modulators that are linked to vascular injury. In this study, we employed tandem LC-MS/MS to identify protein signatures that encompass protein abundance in RASMC treated with BK or leptin followed by systems biology analyses to gain insight into the biological pathways and processes linked to vascular remodeling. In the study, 1837 proteins were identified in control untreated RASMC. BK altered the expression of 72 (4%) and 120 (6.5%) proteins, whereas leptin altered the expression of 189 (10.2%) and 127 (6.5%) proteins after 24 and 48 h, respectively, compared to control RASMC. BK increased the protein abundance of leptin receptor, transforming growth factor-β. On the other hand, leptin increased the protein abundance of plasminogen activator inhibitor 1 but decreased the protein abundance of cofilin. BK and leptin induced the expression of inflammatory cytokines such as tumor necrosis factor alpha (TNF-α) and interleukin-1β (IL-1β) and pathway analysis revealed the activation of mitogen-activated protein kinases (MAPKs) and AKT pathways. The proteome profile in response to BK and leptin revealed mechanistic interplay of multiple processes that modulate inflammation and oxidative stress signals in the vasculature.

## 1. Introduction

Oxidative stress and inflammation are among the primary contributors to cardiovascular diseases (CVDs) such as atherosclerosis and ischemic heart failure [1,2,3,4]. In fact, the induction of oxidative molecules (reactive oxygen and nitrogen species) leads to the initiation of inflammatory responses and the production of inflammatory cytokines [5]. Moreover, oxidative stress has been implicated in developing ischemic heart failure secondary to atherosclerotic plaque occlusion of the coronary arteries [6,7]. In addition, atherosclerosis is defined as low-grade chronic inflammation of the vessel walls of medium- and large-sized arteries [4,8,9]. There are multiple processes for the progression of atherosclerosis such as endothelial dysfunction, vascular smooth muscle cells (VSMC) proliferation and migration, overproduction of extracellular matrix proteins, and accumulation of lipids within macrophages (foam cells) in the sub-endothelial layer in the vessel wall [10]. These processes lead to the hardening of medium- and large-sized arteries at various anatomical sites, thus increasing the risk of developing myocardial infarction and strokes [9]. As a result of vessel stiffness, atherosclerosis modifies vessel tone and blood flow dynamics, which are controlled by the degree of contraction and relaxation of VSMC [11]. Moreover, VSMC proliferation and migration as well as extracellular matrix deposition contribute to atherogenesis by lessening the lumen of the blood vessels [12,13].

Activation of components of the Kallikrein-kinin system (KKS) has been linked to the development of atherosclerosis, and subsequently, CVD [14]. Kallikrein cleavages kininogen (KNG) to release the pro-inflammatory vasoactive peptide bradykinin (BK). The generated BK acts on its constitutively expressed B2-receptors (B2R) in the heart, vasculature, and kidney in an autocrine and or paracrine manner to initiate a multitude of cellular signals that influence vascular and renal function [15,16,17,18,19]. In this regard, we have previously shown that BK can stimulate the generation of reactive oxygen species in VSMC which results in mitogen-activated protein kinase (MAPK) activation and its translocation to the nucleus as well as cell migration and proliferation [20,21]. Moreover, deletion of BK receptor 2 (B2R−/−) attenuated the renal expression of oxidative stress genes in diabetic mice [22].

Another contributor to the development of atherosclerosis is adipose tissue through the secretion of several metabolites, such as leptin [23]. Leptin was shown to be expressed and secreted from extra-adipose tissues [24,25]. Several studies have reported the association between leptin circulatory levels with different CVD risk factors, such as hypertension, extracellular matrix (ECM) deposition, inflammation, and oxidative stress [26,27].

In this study, we employed mass spectrometry to assess the global temporal changes in proteome profile in VSMC stimulated with either BK or leptin. Bioinformatics approaches were employed to characterize the biological processes and signaling networks underlying the development of vascular injury.

## 2. Materials and Methods

### 2.1. Animals

The Institutional Animal Care and Use Committee (IACUC) at the American University of Beirut, in accordance with the National Institutes of Health guide for the care and use of laboratory animals, approved all the experimental protocols with IACUC approval number 17-02-339. Male Sprague-Dawley rats were housed in the animal facility under controlled conditions of light (12 h light, 12 h dark cycle) and temperature (23 °C ± 2 °C).

### 2.2. Primary Cell Extraction

Primary rat aortic smooth muscle cells (RASMC) were cultured from the aorta of male Sprague-Dawley rats according to the modified technique of Majack and Clowes [28]. RASMC were maintained in DMEM medium supplied with 10% FBS and used between passage 2 and 8. Immunocytostaining of smooth muscle α-actin (α-SMA, ab7817, Abcam) was used to verify the identity of the primary extracted vascular cells. Quiescent cell stage was achieved at about 80% confluence by serum starving the cells for 24 h before any treatment. RASMC were incubated with BK 10^−7^ M and leptin 3.1 nM for different time-points. The stimulation of RASMC started at the same time and the treatment was stopped at the specified time of each condition to minimize the batch effect in the experiment. The selection of these concentrations was based on previous studies [29,30,31].

### 2.3. Enzyme-Linked Immunosorbent Assay (ELISA)

The supernatants of the RASMC incubated with BK 10^−7^ M and leptin 3.1 nM for 24 h were collected and used for the measurement of TNF-α using TNF alpha Rat Uncoated ELISA Kit (Cat # 88-7340-88, Invitrogen) according to the manufacturer’s instructions.

### 2.4. Extraction and Tryptic Digestion of Proteins

The extraction and tryptic digestion of the control, BK (24 h or 48 h), and leptin (24 h or 48 h) stimulated RASMC samples were similar to our previous report [15]. The details of the method are discussed in the Supplementary Materials Section.

### 2.5. Liquid Chromatography-Mass Spectroscopy/Mass Spectroscopy Analysis

The tryptic digested proteins were subjected to LC-ESI-MS/MS analysis that is similar to our previous report [15]. The LC-MS/MS experiments for all the samples were run at the same time to minimize the batch effect in the experiments. The analysis was performed using a Dionex Ultimate 3000 nano-LC system interfaced to an LTQ Orbitrap Velos mass spectrometer equipped with a nano-ESI source (Thermo Scientific, San Jose, CA). A detailed description of the method used is provided in the Supplementary Materials Section.

### 2.6. Statistical Analysis and LC-MS/MS Data Analysis

Similar to our previous report [15] LC-MS/MS raw data were searched against the SwissProt database (Rattus) in MaxQuant version 1.5.4.1. Peptides were considered only with a minimum length of 7 amino acids. The false discovery rate (FDR) of 0.01 was used for both peptide and protein identification. Ratio count was set as 2, and both razor and unique peptides were considered for quantification. Protein label-free-quantification (LFQ) intensities produced by MaxQuant were additionally processed using Perseus. Common contaminants and reverse hits for identification of both peptide and protein were removed from the hits. Proteins that were identified in at least 2 replicates from one sample group were reported. Log2 transformed protein LFQ-intensities were subjected to statistical analysis. Two-way ANOVA analysis followed by Tukey’s multiple comparison tests was conducted using GraphPad Prism 7 (GraphPad Software, La Jolla, CA, USA) to determine the significant differentially expressed proteins (*p*-value < 0.05). The ClustVis web tool was utilized to generate the hierarchal clustering of the different time points compared to the control [32].

### 2.7. Systems Biology Assessment

Ingenuity pathway analysis (IPA) was applied to evaluate functional correlations within the various treatment groups. Datasets encompassing protein identifiers (UniProt-KB) and matching expression values (Log2 (Fold change)) of various comparison groups were uploaded. The comparative analyses were as follows: BK 24 h vs. control and BK 48 h vs. control to examine the impact of BK treatment compared to time-matched control, and leptin 24 h vs. control and leptin 48 h vs. control to assess the influence of leptin stimulation compared to time-matched control. Identifier for each protein was plotted to its matching protein entity in the IPA database. All plotted proteins that were differentially expressed with *p* < 0.05 were uploaded against global molecular networks established from data contained in the knowledge base. Gene ontology (GO), diseases, metabolic process, and signaling process were additionally analyzed.

### 2.8. RNA extraction and Real-Time PCR

Total RNA from RASMC was generated by using RiboZol reagent (Amresco) in accordance with the manufacturer’s protocol. The total RNA concentration was measured by Nanodrop 1000 (Thermo Scientific) at 260 nm, and the 260/280 ratio was determined. RNA (1 µg) was reverse transcribed into cDNA and the cDNA amplification reaction was performed using the iQ SYBR green mix kit (Bio-Rad). Primer sequences (TIB Molbiol) for the genes of interest were as follows: rat GAPDH (Forward: GGGGCTCTCTGCTCCTCCCTG, Reverse: CGGCCAAATCCGTTCACACCG), rat leptin (Forward: GAGACCTCCTCCATGTGCTG, Reverse: CATTCAGGGCTAAGGTCCAA), rat leptin receptor (Forward: TGACCACTCCAGATTCCACA, Reverse: CCACTGTTTTCACGTTGCTG), rat IL-1β (Forward: TCCTCTGTGACTCGTGGGAT, Reverse: TCAGACAGCACGAGGCATTT), and TNF-α (Forward: ACCTTATCTACTCCCAGGTTCT, Reverse: GGCTGACTTTCTCCTGGTATG). A standard curve for each target gene was generated by carrying out a series of 10-fold dilutions. PCR was achieved using the CFX96^TM^ Real-Time PCR Detection System (Bio-Rad) programmed for 1 min of denaturation at 98 °C (1 cycle) initially, followed by 40 alternating cycles of 9 s denaturation at 95 °C, 12 s annealing at 55 °C, and 9 s extension at 72 °C followed by one cycle of 10 min extension at 72 °C. Expressed genes were quantified by calculating the ΔΔCt and expressed relative to GAPDH mRNA.

### 2.9. Statistical Analysis

Descriptive statistics were originally performed on the overall mean for every group and for each time point. Data were evaluated graphically for normality using Q-Q plots that define if the data follow the normal distribution as well as numerically using the Shapiro–Wilk test for normality. Independent t-test was performed if normality was met to compare the group means and the reported *p*-values were centered on the homogeneity test. The nonparametric Mann–Whitney U test was performed as an alternate method when the normality of the data could not be assumed. Data are expressed as mean ± SE, and significance was considered at *p* < 0.05 level.

## 3. Results

### 3.1. RASMC Proteomic Analysis

Proportional proteomic profiling of RASMC protein abundance in response to either BK or leptin time point stimulation was done using LC-ESI-MS/MS followed by MaxQuant analysis of the generated protein spectra. Embracing this method, 1837 diverse proteins were recognized in the control samples of RASMC. As shown in the LFQ file (Figure S1), the distribution of the identified protein showed a bell-shape indicating the normal distribution of the identified proteins.

### 3.2. Effect of BK Stimulation on the Proteome Profile in RASMC

**Figure 1:**
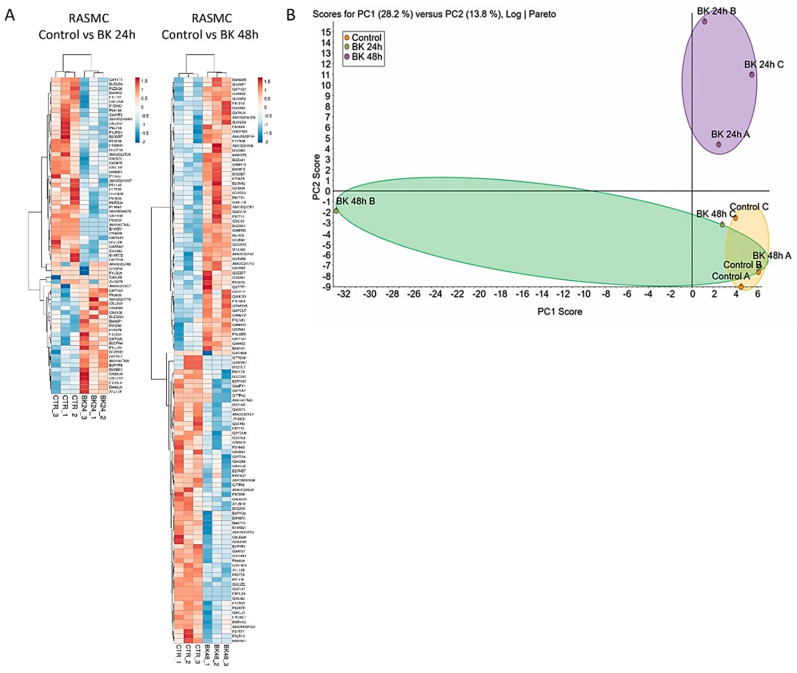
Characteristics of proteomic of bradykinin (BK) stimulated rat aortic smooth muscle cell (RASMC). (**A**). Hierarchical clustering (heat maps) of protein expression profiles in the BK (24 h or 48 h) stimulated RASMC compared to control. The downregulated proteins are represented in blue, whereas the upregulated proteins are represented in red. The level of the protein regulation is reflected in the color intensity and the accession numbers of the proteins are listed to the right side of the heat maps. The dendrogram shows the clustering of the proteins and the samples with each other. (**B**). Principal component analysis (PCA) of the BK-stimulated RASMC samples. The axis of the principal components is depicted according to the protein expression. The control samples are enclosed by the yellow oval, the BK 24 h stimulated samples are enclosed by the violet oval, and the BK 48 h stimulated samples are enclosed by the green oval.

The effect of time points BK-stimulation (24 h or 48 h) of RASMC showed a temporal profile of protein expression in response to BK as illustrated in the heat maps (Figure 1A and Tables S1 and S2). Although there were more than 1800 identified proteins in the control samples, 72 (4%) proteins were significantly modified after 24 h of BK stimulation with *p* < 0.05, and of these 28 were upregulated and 44 were downregulated. Among the modified proteins after 24 h stimulation with BK, leptin receptor (LEPR) was upregulated (1.83 ± 0.12-fold, *p* = 0.006) compared to the control samples. In addition, 120 (6.5%) proteins were significantly modified after 48 h of BK stimulation with *p* < 0.05, and the number of the upregulated and downregulated proteins was almost the same (list of proteins in Tables S1 and S2). Among the modified proteins after 48 h stimulation with BK, transforming growth factor beta-1-induced transcript 1 protein (TGFB1I1) was upregulated (1.28 ± 0.03-fold, *p* = 0.022) compared to the control samples.

### 3.3. Principal Component Analysis (PCA)

To gain an understanding of the global variations occurring in the different stimulated groups, we employed a multivariate data analysis, PCA, that is utilized to scheme and transmute the comprehensive changes in large datasets into two-dimensional plots to illustrate the commonness of the alterations amongst the dissimilar groups. PCA plots of the BK-treated samples showed an effectual separation amid the various groups, signifying that the temporal BK treatments generated distinctive proteomic profiles. Moreover, we identified a commonality among the PCA plot of the BK (48 h) and control samples (Figure 1B).

### 3.4. Network and Pathway Analysis of Proteomic Profiles

**Figure 2:**
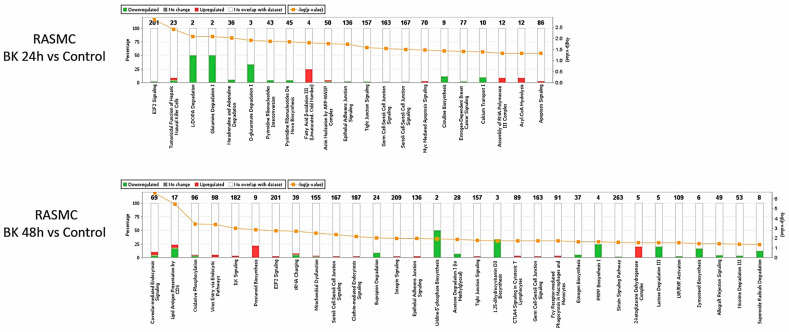
The regulated canonical pathways in response to BK stimulation in RASMC samples. Altered canonical pathways in response to the protein profile in the BK 24 h and 48 h stimulated vs. control samples. Each altered canonical pathway is represented in a bar graph. The downregulated proteins are represented by green on the graph, whereas the upregulated proteins are represented by red on the graph.

The significantly modified proteins in the BK time-point stimulations were analyzed through IPA to depict the interaction between the modified proteins, to suggest partner proteins to link the modified proteins, and to predict the effect of the modified proteins on their neighboring proteins pertaining to their activity status. We first depicted the effect of the BK time-point stimulations on the activity of the canonical pathways (Figure 2). We noticed that there are common pathways altered between the two BK time-point stimulations such as EIF2 signaling, epithelial adherens junction signaling, pyrimidine ribonucleotides de novo biosynthesis, germ cell-Sertoli cell junction signaling, and Sertoli cell-Sertoli cell junction signaling. However, the modification of these pathways was in the opposite direction of regulation between the two time-point stimulations.

Network analysis of BK stimulation for 24 h compared to control (Figure S2) showed that sarcoplasmic/endoplasmic reticulum calcium ATPase 2 isoform b (Atp2a2) (0.87±0.0.03-fold, *p* = 0.036) was downregulated by the BK stimulation and connected to the inhibition of superoxide dismutase (SOD2) and nuclear factor kappa B (NFκB) complex and the activation of tumor necrosis factor (TNF) and Jnk. These processes influenced many toxicity functions such as oxidative stress, mitochondrial dysfunction, and NRF2-mediated oxidative response.

**Figure 3:**
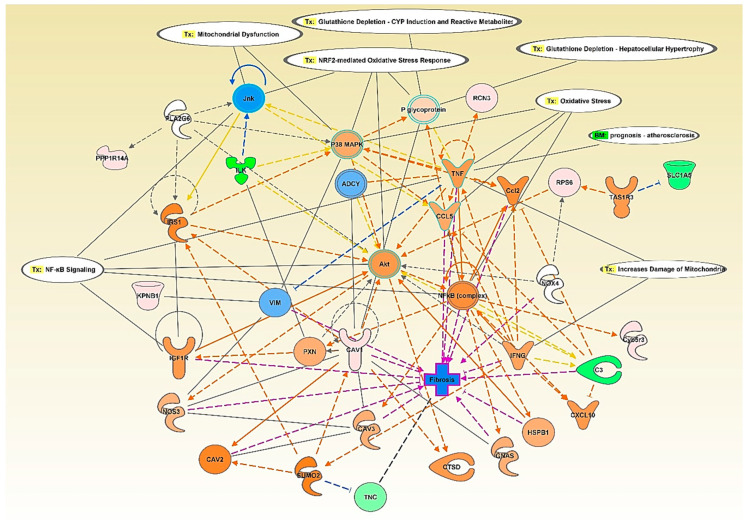
Ingenuity pathway analysis (IPA) network analysis of the modified proteins in response to BK 48 h stimulation relative to control in RASMC. The top toxicity (Tox) functions linked to the altered proteins are oxidative stress, mitochondrial dysfunction, increased damage of mitochondria, NRF2 mediated stress response, and glutathione depletion. Red indicates an upregulated protein, green indicates a down-regulated protein, orange indicates an activated protein, and blue indicates an inhibited protein. The color intensity represents the level of expression.

Network analysis of BK stimulation for 48 h compared to control (Figure 3) showed that caveolin-1 alpha isoform (Cav1) (1.74 ± 0.14-fold, *p* = 0.047) was upregulated and connected to the activation of TNF, interleukin-1β (IL-1β), P glycoprotein insulin-like growth factor 1 receptor (IGF1R), Akt, cathepsin D (CTSD), insulin receptor substrate 1 (IRS1), nitric oxide synthase 3 (NOS3), and paxillin (PXN). In addition, this network showed that the altered proteins influenced many cardiovascular toxicity functions such as mitochondrial dysfunction, oxidative stress, NRF2-mediated oxidative response, glutathione depletion, and transforming growth factor beta (TGF-β) signaling.

Furthermore, BK stimulation for 48 h induced the expression of TGFB1I1 that is connected to the stimulation of TGF-β, serpin family E member 1 (SERPINE1), SMAD family member 3 and 7 (SMAD3 and 7), latent transforming growth factor beta binding protein 1 and 2 (LTBP1 and 2), and collagen Alpha 1 (Figure S3).

**Figure 4:**
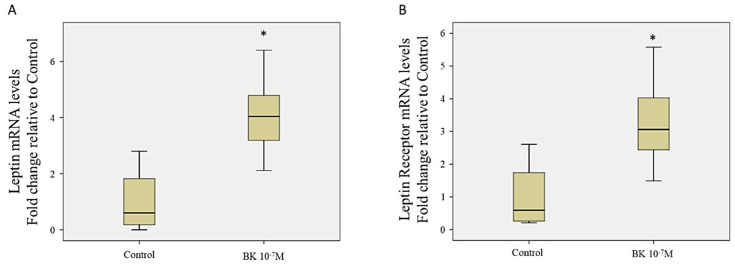
Assessment of leptin and leptin receptor expression. (**A**). mRNA level assessment of leptin mRNA by RT-qPCR in response to BK 24 h relative to non-stimulated controls. Boxplots are representative of 4 different repeats. (**B**). mRNA level assessment of leptin receptor mRNA by RT-qPCR in response to BK 24 h relative to non-stimulated controls. Boxplots are representative of 5 different repeats. * *p* < 0.05 BK vs. control.

To confirm the findings of the proteomic results, we assessed the mRNA levels of leptin and leptin receptor (LeptR) in BK time-point stimulated RASMC. Our results showed that BK stimulated the mRNA expression of both leptin (4.1 ± 0.73-fold, *p* = 0.027 BK 24 h vs. control) and LeptR (3.32 ± 0.7-fold, *p =* 0.05 BK 24 h vs. control) after 24 h stimulation (Figure 4). Moreover, we assessed the protein expression of LeptR in response to the time-point stimulations by BK. Our results showed that BK induced the expression of LeptR after 24 h stimulation compared to the control (Figure S4).

### 3.5. Effect of Leptin Stimulation on the Proteome Profile in RASMC

**Figure 5:**
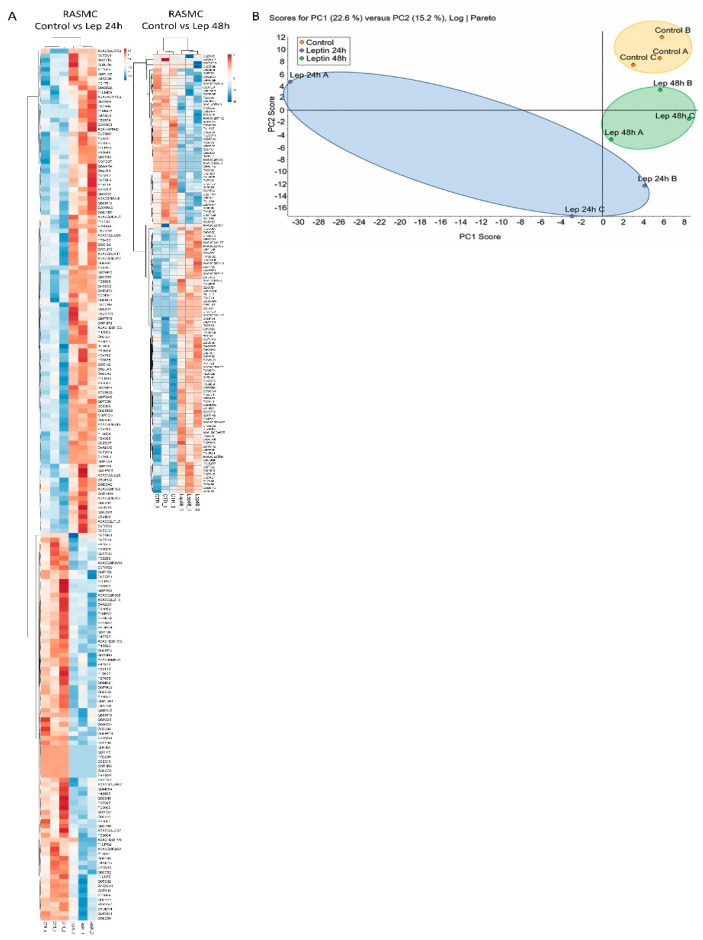
Characteristics of proteomic of leptin-stimulated RASMC. (**A**)**.** Hierarchical clustering (heat maps) of protein expression profiles in the leptin- (24 h or 48 h) stimulated RASMC compared to control. The downregulated proteins are represented in blue, whereas the upregulated proteins are represented in red. The level of the protein regulation is reflected in the color intensity and the accession numbers of the proteins are listed to the right side of the heat maps. The dendrogram shows the clustering of the proteins and the samples with each other. (**B**)**.** Principal component analysis (PCA) of the leptin-stimulated RASMC samples. The axis of the principal components is depicted according to the protein expression. The control samples are enclosed by the yellow oval, the leptin 24 h stimulated samples are enclosed by the blue oval, and the leptin 48 h stimulated samples are enclosed by the green oval.

One of the significant observations in the BK stimulation results was the upregulation of the LeptR in the proteomic and expression results. We sought to assess the effects of LeptR in RASMC by stimulating these cells with leptin. The effect of time point leptin-stimulations (24 h or 48 h) of RASMC showed a temporal profile of protein expression in response to leptin as illustrated in the heat maps (Figure 5A and Tables S3 and S4). Although there were more than 1800 identified proteins in the control samples, 189 (10.2%) proteins were significantly modified after 24 h of leptin stimulation with *p* < 0.05, and of these 108 were upregulated and 81 were downregulated. In addition, 127 (6.5%) proteins were significantly modified after 48 h of leptin stimulation with *p* < 0.05, and of these 78 were upregulated and 49 were downregulated.

**Figure 6:**
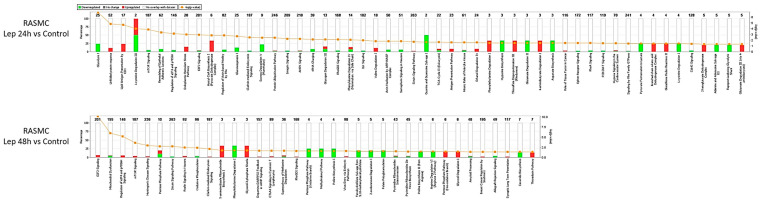
The regulated canonical pathways in response to leptin stimulation in RASMC samples. Altered canonical pathways in response to the protein profile in the leptin 24 h and 48 h stimulated vs. control samples. Each altered canonical pathway is represented in a bar graph. The downregulated proteins are represented by green on the graph, whereas the upregulated proteins are represented by red on the graph.

Moreover, we employed PCA to depict the commonness of the changes between the groups. PCA plots of the leptin-stimulated samples showed an efficient separation between the various groups, demonstrating that the leptin time point treatments have distinct temporal effects on the expression of the proteins in RASMC (Figure 5B). Furthermore, the modification of the canonical pathway in response to leptin time-point stimulations was assessed (Figure 6). Our results showed that there are common pathways altered between the two leptin time point stimulations such as mTOR signaling, regulation of eIF4 and p70S6K signaling, EIF2 signaling, RhoGDI signaling, and phenylethylamine degradation I.

However, the modifications of these pathways were in the opposite direction of regulation between the two time point stimulations. It is noteworthy that there were common modified pathways between the BK and leptin-stimulated samples, among which were actin nucleation by ARP-WASP complex, ILK signaling, mitochondrial dysfunction, clathrin-mediated endocytosis signaling, integrin signaling, and EIF2 signaling.

**Figure 7:**
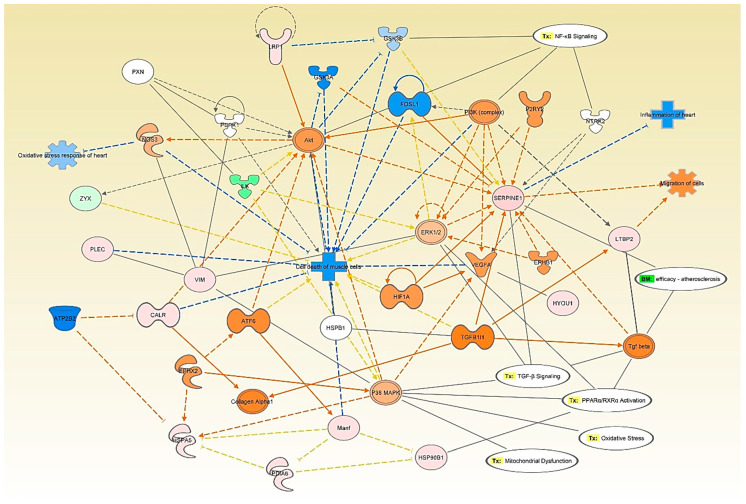
IPA network analysis of the modified proteins in response to leptin 24 h stimulation relative to control in RASMC. The top Tox functions related to the modified proteins are oxidative stress, mitochondrial dysfunction, TGF-β signaling, and NFκB signaling. Red indicates an upregulated protein, green indicates a down-regulated protein, orange indicates an activated protein, and blue indicates an inhibited protein. The color intensity represents the level of expression.

Network analysis of the effect of leptin stimulation for 24 h compared to control (Figure 7) showed upregulation of plasminogen activator inhibitor 1 (Serpine1) (1.65 ± 0.082-fold, *p* = 0.029), vimentin (Vim) (1.22 ± 0.08-fold, *p* = 0.047), and prolow-density lipoprotein receptor-related protein 1 precursor (Lrp1) (1.09 ± 0.03-fold, *p* = 0.022). On the other hand, leptin stimulation for 24 h compared to control showed the downregulation of cofilin-1 (Cfl1) (0.77 ± 0.06-fold, *p* = 0.009), peroxiredoxin-6 (Prdx6) (0.69 ± 0.13-fold, *p* = 0.023), tropomyosin 1, alpha (Tpm1) (0.42 ± 0.69-fold, *p* = 0.035), and myosin light chain 10 (Myl10) (0.12 ± 0-fold, *p* = 0.003). The upregulation of SERPINE1 was connected to TGF-β signaling and NFκB signaling through the activation of AKT, ERK1/2, and PI3K among others.

**Figure 8:**
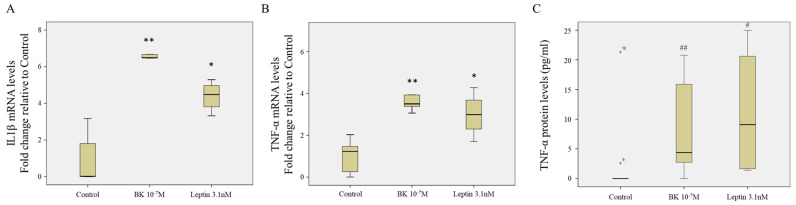
Assessment of the expression of IL-1β and TNF-α. (**A**). mRNA level assessment of IL-1β gene by RT-qPCR in response to BK and leptin 24 h relative to non-stimulated controls. Boxplots are representative of 5 different repeats. ** *p* < 0.01 BK vs. control and * *p* < 0.05 leptin vs. control. (**B**). mRNA level assessment of TNF-α gene by RT-qPCR in response to BK and leptin 24 h relative to non-stimulated controls. Boxplots are representative of 5 different repeats. ** *p* < 0.01 BK vs. control and * *p* < 0.05 leptin vs. control. (**C**). Protein level assessment of TNF-α protein (pg/mL) by ELISA in response to BK and leptin 24 h. Boxplots are representative of 11 repeats of control, 8 repeats of BK, and 4 repeats of leptin. The two asterisks (numbered 9 and 10) above the control average represent two outlier values for the levels of the TNF-α in the control samples as indicated by the conducted analysis. ^##^ *p* = 0.008 of BK vs. control adjusted for multiple comparisons and ^#^ *p* = 0.013 of leptin vs. control adjusted for multiple comparisons.

Upon analyzing our data, IPA suggested the activation of interleukin-1 β (IL-1β) and tumor necrosis factor- α (TNF-α) downstream to the 24 h stimulation by both BK and leptin (Figure 3 and Figure S5). Therefore, we assessed the mRNA levels of IL-1β and TNF-α and the protein levels of TNF-α in response to 24 h stimulation by BK and leptin. Our results showed that both BK and leptin induced the gene expression of IL-1β (6.63 ± 0.26-fold, *p* = 0.009 BK 24 h vs. control, and 4.06 ± 0.28-fold, *p* = 0.014 leptin 24 h vs. control) and the gene expression of TNF-α (4.15 ± 0.68-fold, *p* = 0.009 BK 24 h vs. control, and 3.34 ± 0.36-fold, *p* = 0.027 leptin 24 h vs. control) after 24 h stimulation of RASMC (Figure 8A,B). Moreover, our results showed that both BK and leptin induced the protein levels of TNF-α (8.35 ± 2.92 pg/mL, *p* = 0.008 BK 24 h vs. control, and 11.13 ± 5.76 pg/mL, *p* = 0.013 leptin 24 h vs. control) after 24 h stimulation of RASMC (Figure 8C).

Finally, leptin stimulation for 48 h compared to control (Table S4) showed that thioredoxin reductase 1 (Txnrd1) (8.13 ± 0.23-fold, *p* = 0.0006), collagen type IV alpha 2 chain (Col4a2) (1.97±0.97-fold, *p* = 0.015), and tyrosine-protein kinase HCK (Hck) (1.40 ± 0.11-fold, *p* = 0.021) were upregulated. Moreover, leptin receptor overlapping transcript-like 1 (Leprotl1) (0.76 ± 0.14-fold, *p* = 0.039), collagen alpha-1(VIII) chain precursor (Col8a1) (0.73±0.037-fold, *p* = 0.009), tropomyosin 1, alpha (Tpm1) (0.56 ± 0.06-fold, *p* = 7.9788E-05), and fibrillin-1 precursor (Fbn1) (0.31 ± 0.27-fold, *p* = 0.035) were downregulated. The activation of TNF and Jnk and the inhibition of the SOD2 were connected to NRF2-mediated oxidative response, mitochondrial dysfunction, and oxidative stress (Figure S6).

## 4. Discussion

Oxidative stress continues to be among the primary contributors to a multitude of diseases, such as atherosclerosis and CVD. The exact mechanisms that contribute to risk of developing atherosclerosis and/or CVD are not well defined. Employing proteomic approaches to study the global protein abundance profile in different pathologies has demonstrated the strength of these approaches in the discovery of biomarkers of diseases or their etiology or stage [33,34]. In this study, the LC-MS/MS technique was employed to assess the global protein profile in primary RASMC stimulated for two time points (24 and 48 h) with either BK or leptin. Moreover, bioinformatics analyses were applied to link the modified proteins and to highlight the biological processes and networks that they are linked to.

Our data showed that time is a factor affecting the stimulation of RASMC by BK or leptin, as evidenced by the number of modified proteins, compared to control. For instance, there were more proteins modified after 48 h stimulation by BK compared to the 24 h stimulated samples. Moreover, the altered canonical pathways of BK time points showed different modified pathways between the two conditions, with some common pathways that are primarily related to the cell junction signaling. In addition, the PCA plots of BK and leptin stimulation indicated a temporal modification of the protein in response to each of them.

To further comprehend the effects of BK time points stimulation in RASMC, we linked the biological processes and the development of diseases with the modified proteins. For instance, we observed an increase in TGF-β post-BK stimulation for 48 h. This regulation of TGFβ downstream of BK stimulation is in line with our previous reports. We have previously shown that BK promotes vascular fibrosis through the induction of ECM protein expression and secretion of tissue inhibitor of metalloproteinase 1 (TIMP1) via the TGFβ pathway [35].

Moreover, we have also observed an increase in the expression of LeptR after 24 h BK stimulation and a decrease in vasodilator-stimulated phosphoprotein (VASP) after 24 and 48 h of BK stimulation. This is the first report in the literature to describe the regulation of leptin receptor and VASP in response to BK. In one of the reports, Nevelsteen et al. studied the involvement of leptin or leptin receptor in the BK-induced vasorelaxation in aortic rings. They demonstrated that the genetic ablation of leptin or its receptor aggravated the endothelial dysfunction and dampened the vascular reactivity of BK [36]. As for the VASP activity, it has been reported that VASP is a requirement for endothelial cells to promote vasodilatation in mesenteric arteries [37]. Our data showed that BK-induced a down-regulation of VASP expression in RASMC, which is an indicator of the aggravation of vasodilatation impairment upon the direct effect of BK on RASMC.

We further investigated the BK-induced upregulation of LeptR in RASMC by examining the protein profile of RASMC stimulated by leptin. We observed that the temporal pattern of modified proteins in response to leptin was different. For instance, stimulating RASMC with leptin for 24 h modified a larger number of proteins than at the 48 h time point. Furthermore, the altered canonical pathways of leptin time points showed different modified pathways between the two conditions. For example, leptin 24 h stimulated the regulation of actin-based motility by Rho among other pathways. The involvement of Rho-A signaling downstream of the leptin receptor is well established in the literature [38]. Moreover, leptin 48 h stimulation showed a different pattern of altered pathways. Furthermore, there were commonly altered pathways between the two time points of leptin stimulation that are primarily related to mTOR signaling and EIF2 signaling. The relationship between leptin and mTOR signaling, and its implication in pathophysiological conditions such as cardiovascular diseases, has been previously discussed and reviewed in the literature [39]. In addition, Fazolini et al. have shown that mTOR signaling downstream of leptin receptor contributes to obesity-dependent colorectal cancer susceptibility [40].

Moreover, we observed an induction of collagen IV alpha 2 protein at the two time points of leptin treatment. Previous reports showed the importance of collagen IV protein to transduce the mechanical stretching effects from the extracellular matrix area into an intracellular signal transduction. Additionally, these reports showed that this mechano-transduction communication is RhoA dependent in RASMC. Furthermore, the increase in collagen IV expression is reflective of an increase of stress fiber and focal adhesion rearrangement in RASMC, and the rearrangement of the ECM protein components in the development of vascular fibrosis [35,38,41].

Furthermore, leptin stimulation showed a down-regulation in the expression of cofilin-1 and peroxiredoxin-6 expression. The downregulation of cofilin-1 goes in line with the well-established role of leptin in actin remodeling. Leptin activates RhoA-LIMK pathway to phosphorylate and inhibit the activity of cofilin-1. Active, non-phosphorylated, cofilin-1 catalyzes the actin depolarization from the filamentous form into the globular form [42]. Leptin, upon inhibiting cofilin-1, preserves the filamentous type of actin aiding the functions of actin polymerization in mobility and proliferation [43]. The downregulation of cofilin-1 by leptin is another pathway by which leptin regulates actin remodeling in RASMC. On the other hand, the downregulation of peroxiredoxin-6 is a mechanism of oxidative stress promotion by leptin in RASMC [44]. This mechanism is not well established and warrants further investigation.

Among the remarkable findings of this study are the downregulation of tropomyosin, the involvement of PAI1 (SERPINE1), the involvement of IL-1β and TNF-α, and the involvement of common signaling pathways downstream of both BK and leptin stimulation.

As for the regulation of tropomyosin and PAI, King-Briggs et al. found a reduction of RASMC tropomyosin caused by balloon-induced carotid injury. In addition, these studies have linked vascular injury to the TGFβ pathway [45]. Furthermore, we previously reported that PAI1 levels are positively associated with plasma prekallikrein levels in type 1 diabetic subjects and that plasma prekallikrein was shown to be significantly associated with carotid intima-medial thickness and its progression in type 1 diabetic subjects [46]. Our results showed that leptin directly upregulates the expression of PAI1, and systems biology analysis suggested the activation of PAI1 downstream of BK 48 h stimulation. In addition, we noticed that Hck was commonly upregulated in BK 48 h time point and both leptin time points. Hck was shown to be involved in promoting the formation of atherosclerotic plaque by activating endothelial cells and monocytes adhesion and migration [47]. Furthermore, Page et al. reviewed the involvement of Hck in the induction of inflammation and cardiovascular diseases [48].

Besides, the network analyses have indicated the involvement of MAPKs (ERK1/2, Jnk, and p38) and AKT pathways as a junction downstream of BK and leptin stimulation. Our previous reports pinpointed the significance of the activation of MAPK pathways to relay the signaling cascade of BK leading to glomerular injury and vascular fibrosis [18,35]. Although reports investigating the signaling activity of AKT downstream of BK are scarce, Srivastava et al. showed that BK activates AKT/ERK/NFκB signaling axis in osteoblasts and Han et al. showed that the activation of AKT leads to the induction of inflammatory cytokines, namely IL-1β and TNF-α Han et al. have shown thagt in microglia [49,50]. Furthermore, our network analyses and gene expression assessment showed a link between BK and leptin stimulation and the induction of the inflammatory cytokines, namely IL-1β and TNF-α, and signaling pathways. The interplay of these mechanisms warrants further investigation to decipher the mediators and events of activation and to explore potential therapeutic strategies or interventions.

## 5. Conclusions

Taken together, our global proteome profile in response to BK stimulation showed induction in leptin receptor, oxidative stress proteins, and inflammatory protein expression. On the other hand, leptin stimulation induced ECM proteins and reduced actin-remodeling proteins. Collectively, these findings suggest that BK stimulates and activates leptin-signaling leading to a multitude of altered cellular pathways that can modulate vascular biology. Further studies are needed to delineate the mechanism(s) linking BK to leptin signaling and to define their functional roles in promoting vascular inflammation and oxidative stress.

## Supplementary Materials

The following are available online at https://www.mdpi.com/2076-3921/9/12/1251/s1: Figure S1: Protein label-free-quantification (LFQ) intensities of the study samples. Figure S2: IPA network analysis of the modified proteins. Figure S3: IPA network analysis of the modified proteins. Figure S4: Immunocytochemistry staining assessing the expression of LeptR (FITC, Green). Figure S5: IPA network analysis of the modified proteins in response to leptin 24 h stimulation relative to control in RASMC. Figure S6: IPA network analysis of the modified proteins in response to leptin 48 h stimulation relative to control in RASMC. Table S1: Comparative list of proteins in BK stimulation for 24 h compared to controls. Table S2: Comparative list of proteins in BK stimulation for 48 h compared to controls. Table S3: Comparative list of proteins in leptin stimulation for 24 h compared to controls. Table S4: Comparative list of proteins in leptin stimulation for 48 h compared to controls.
