# Supplementary material for: Vascular Cells Proteome Associated with Bradykinin and Leptin Inflammation and Oxidative Stress Signals
Source: Antioxidants (Basel). 2020 Dec 9;9(12):1251. doi: 10.3390/antiox9121251 (PMC7764689; doi:10.3390/antiox9121251)
Supplement: Supplementary file 1 [file antioxidants-09-01251-s001.pdf]

Supplementary Material

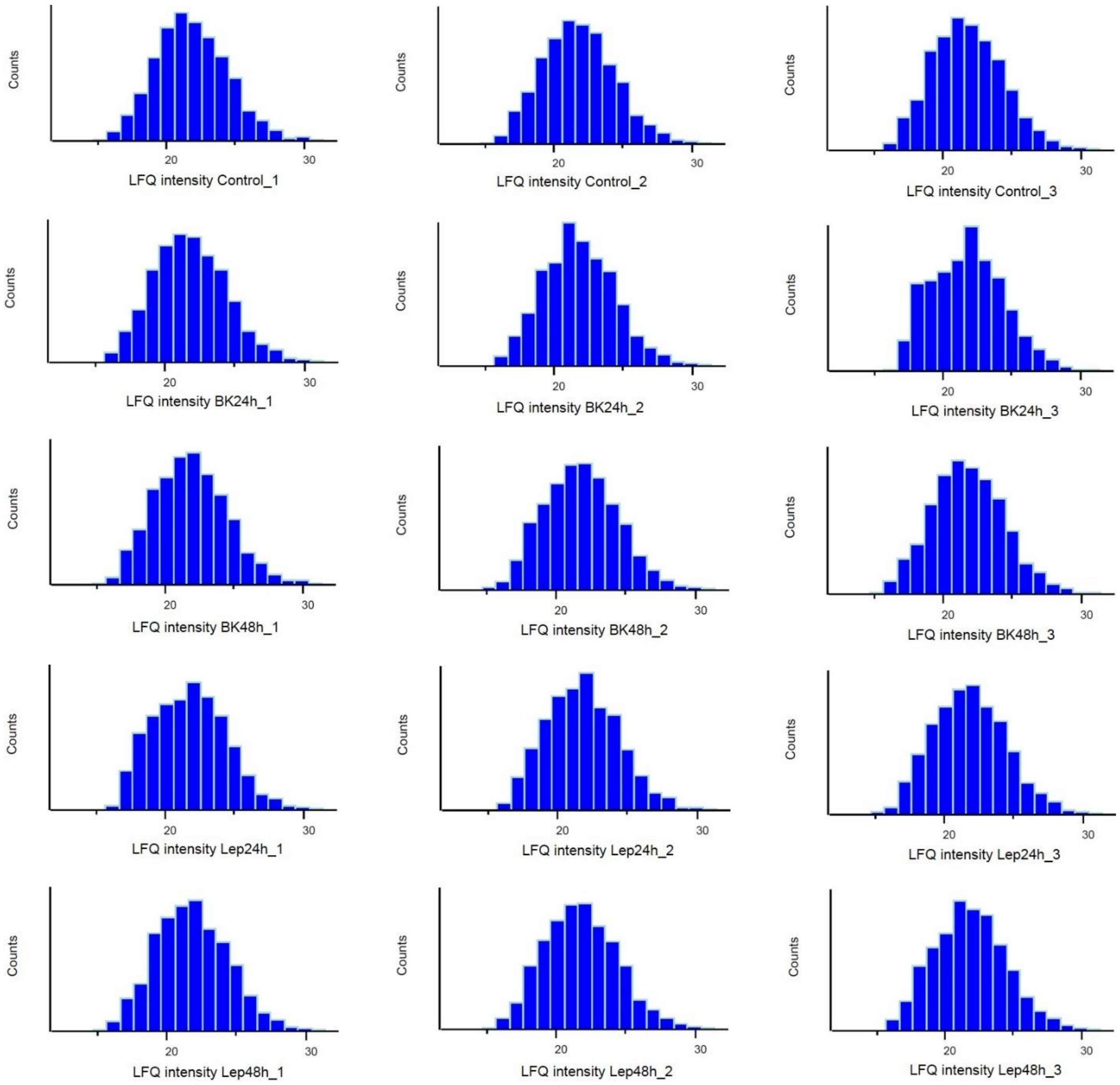

Figure 1. Protein label-free-quantification (LFQ) intensities of the study samples. The bar graphs show that the identified proteins are normally distributed in all the samples.

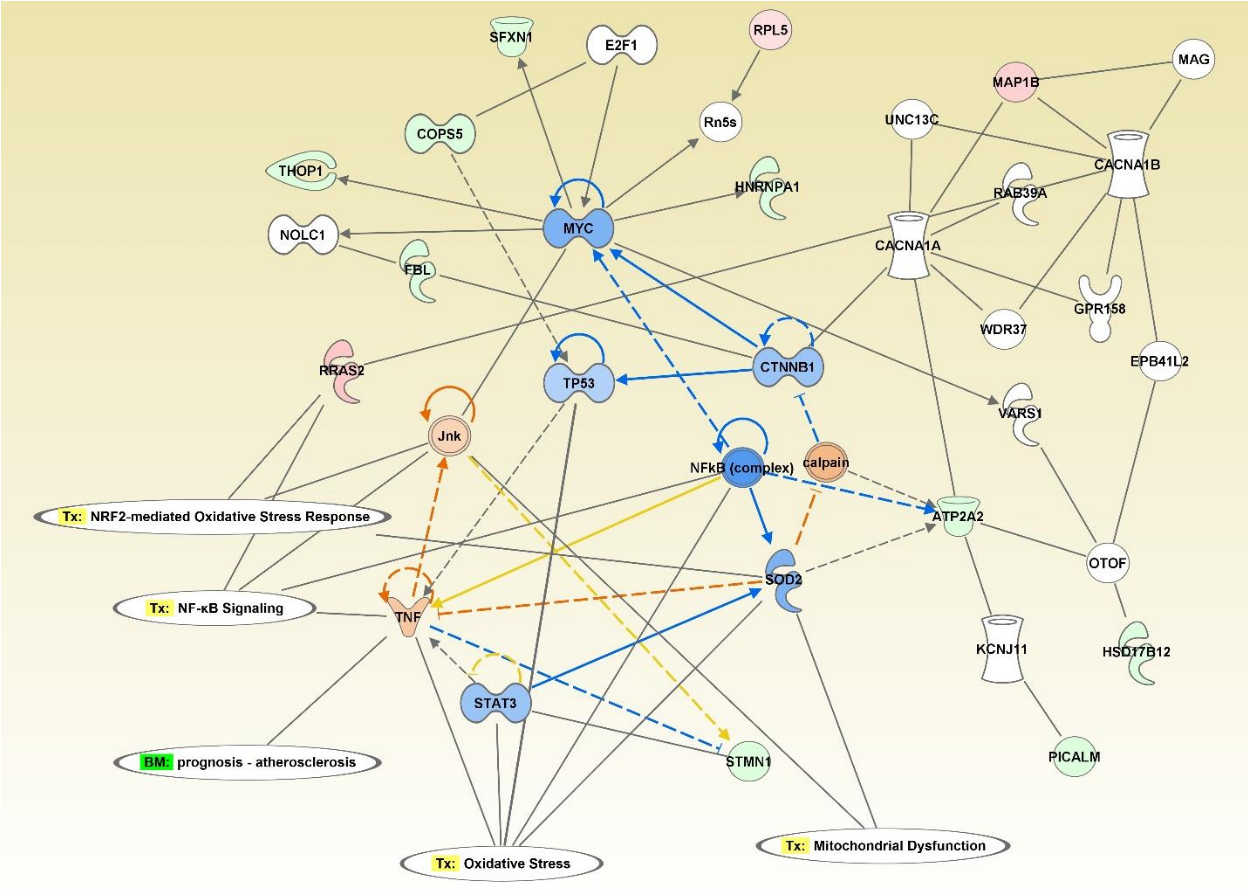

Figure 2. IPA network analysis of the modified proteins in response to BK 24h stimulation relative to control in RASMC. Involvement of TNF, Jnk, and SOD2 downstream of BK stimulation.

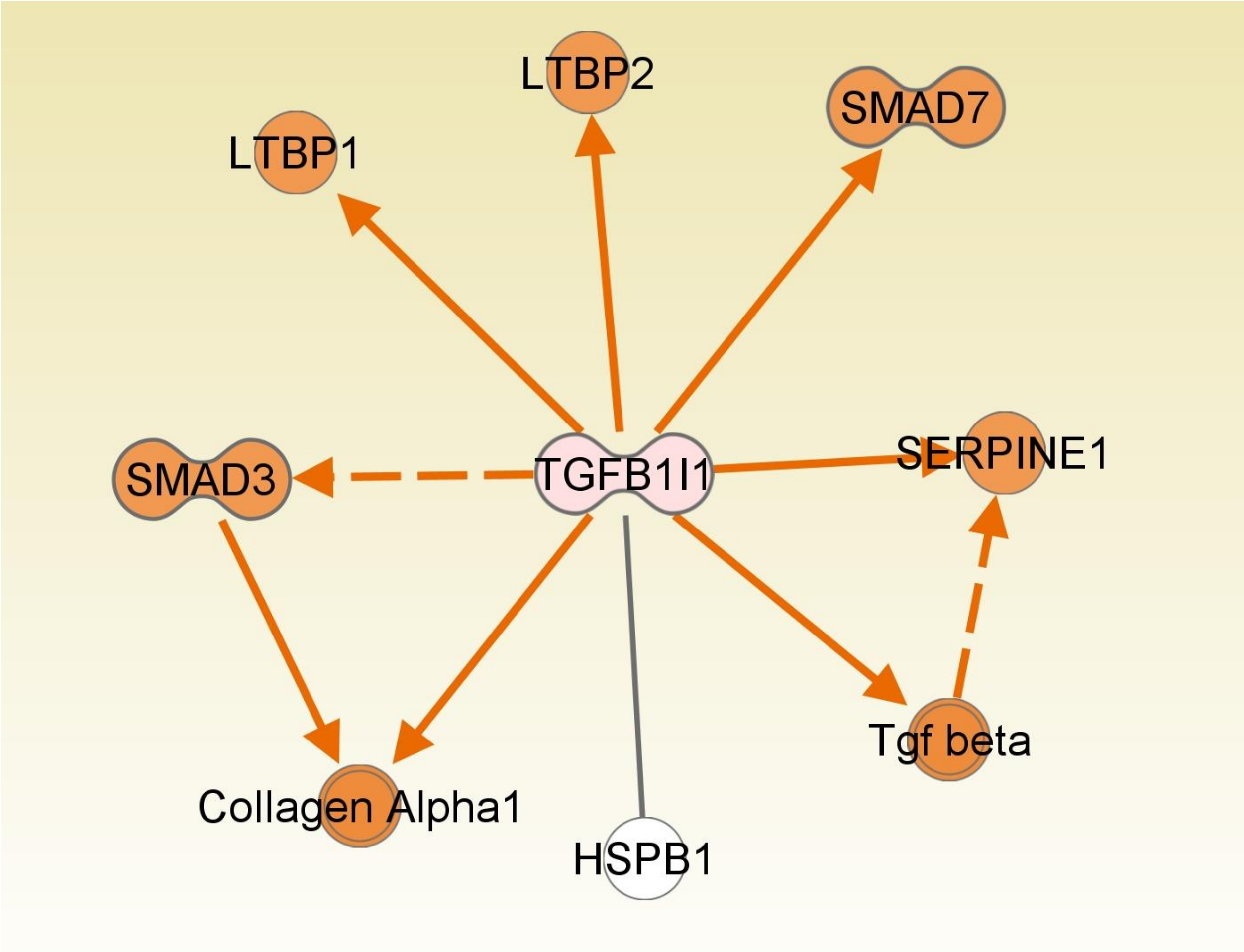

Figure 3. IPA network analysis of the modified proteins in response to BK 48h stimulation relative to control in RASMC. Activation of SERPINE1 downstream of BK stimulation.

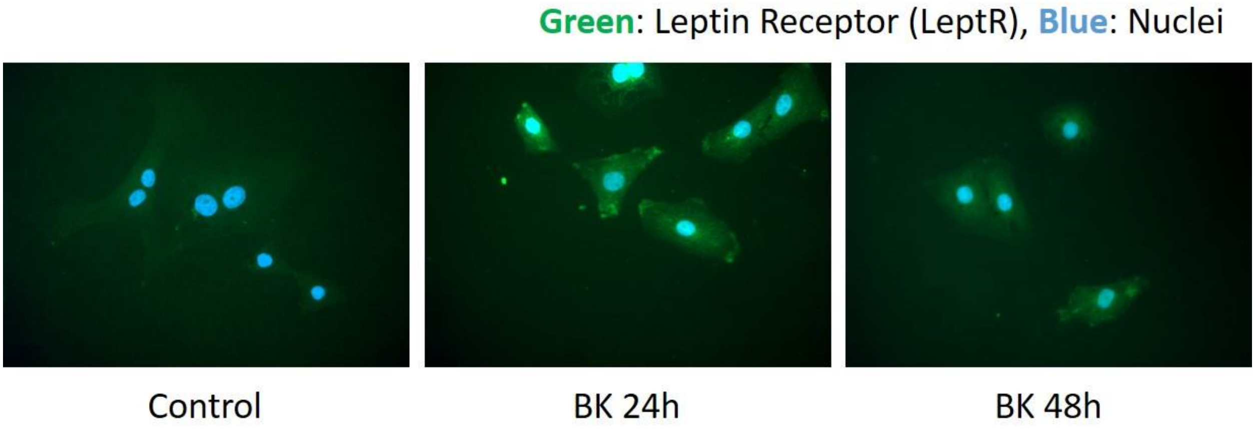

Figure 4. Immunocytochemistry staining assessing the expression of LeptR (FITC, Green) in response to BK time point stimulations in RASMC.

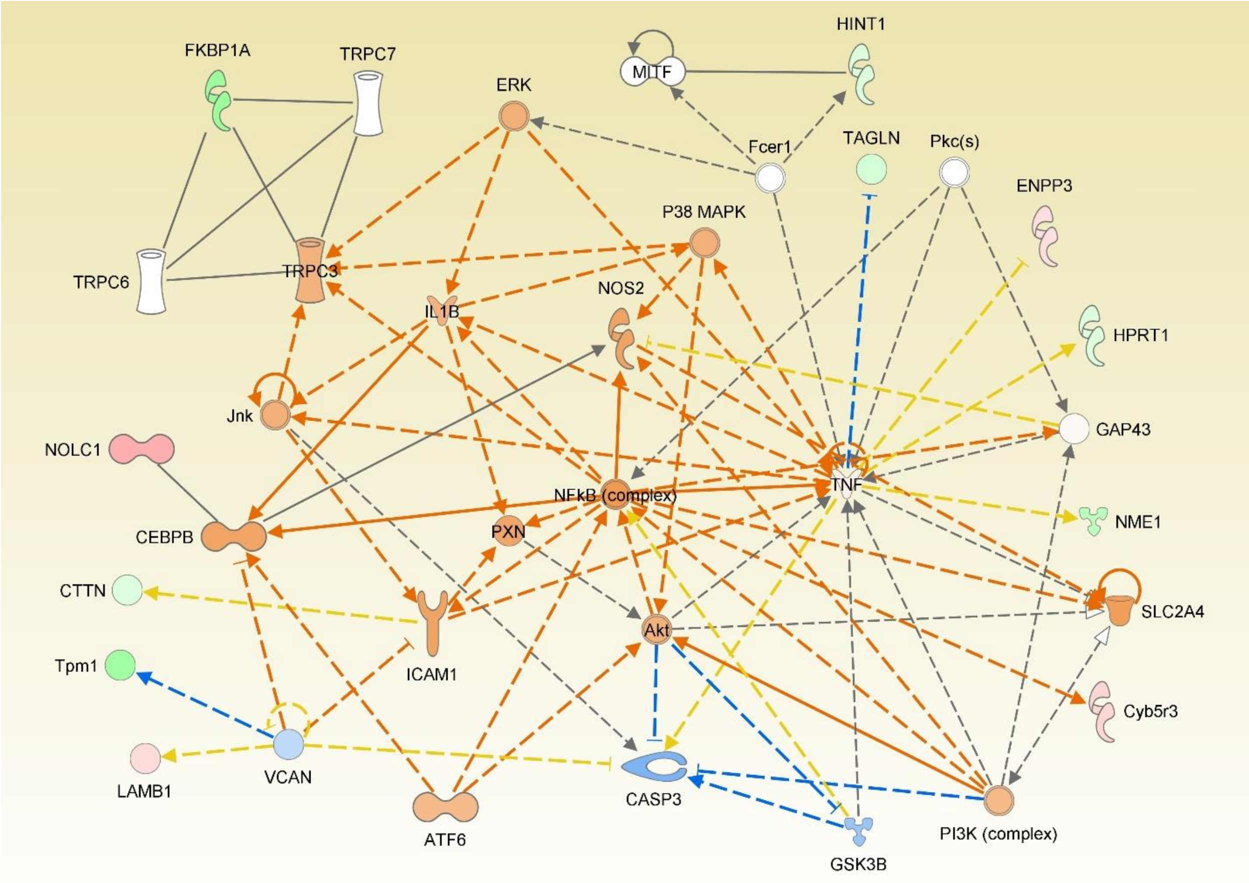

Figure 5. IPA network analysis of the modified proteins in response to Leptin 24h stimulation relative to control in RASMC. Involvement of IL1β and TNF downstream of Leptin stimulation.

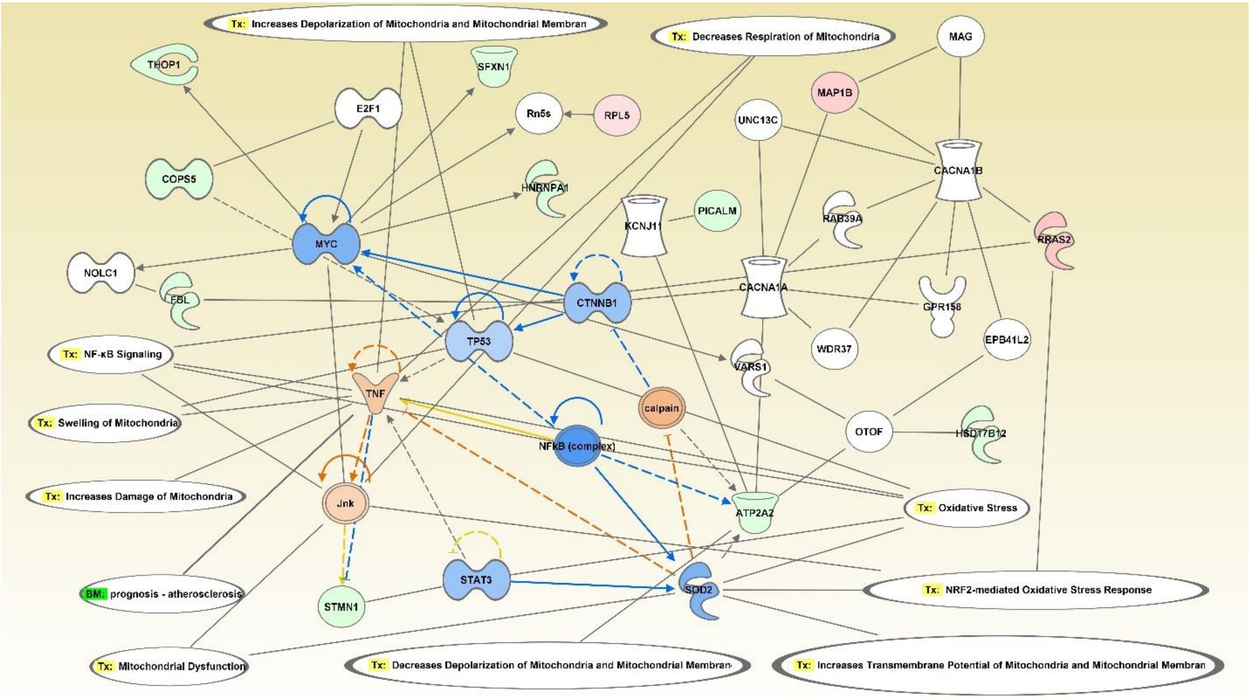

Figure 6. IPA network analysis of the modified proteins in response to Leptin 48h stimulation relative to control in RASMC. Involvement of NFκB, TNF, Jn, and SOD2 downstream of Leptin stimulation.

Table 1. Comparative list of proteins in the BK-stimulated for 24h compared to controls.

Number	Accession	Name	q-value	Fold change
**1**	B2RYL3	Tmem119 - Transmembrane protein 119 precursor	0.002	6.18
**2**	A0A140TAI9	Leprotl1 - Leptin receptor overlapping transcript-like 1	0.006	1.83
**3**	Q9JLS8	Leprot - Leptin receptor gene-related protein	0.040	1.82
**4**	D4A5L9	LOC690675 - Electron carrier protein	0.033	1.66
**5**	Q5BJU0	Rras2 - Ras-related protein R-Ras2	0.044	1.62
**6**	B2RYP6	Luc7l2 - LUC7-like 2	0.011	1.56
**7**	A1L1J9	Lmf2 - Lipase maturation factor 2	0.020	1.51
**8**	F1LRL9	Map1b - Microtubule-associated protein 1B	0.045	1.50
**9**	D4AB03	Fam120a - Constitutive coactivator of PPAR-gamma-like protein 1	0.007	1.44
**10**	Q499N6	Ubxn1 - UBX domain-containing protein 1	0.023	1.40
**11**	Q5HZY0	Ubxn4 - UBX domain-containing protein 4	0.036	1.39
**12**	D4A5F1	Pkd2 - Polycystin-2	0.036	1.38
**13**	Q6P7Q1	Bre - BRCA1-A complex subunit BRE	0.042	1.35
**14**	Q5U2V8	Emc3 - ER membrane protein complex subunit 3	0.003	1.35
**15**	F2Z3S5	Rbpms - RNA-binding protein with multiple-splicing	0.050	1.32
**16**	A0A0G2JZ48	Tmf1 - TATA element modulatory factor 1	0.012	1.31
**17**	Q5XIG8	Strap - Serine-threonine kinase receptor-associated protein	0.024	1.30
**18**	A0A0G2JYC7	Hdgfrp2 - Uncharacterized protein	0.035	1.30
**19**	F1LUA1	Eea1 - Early endosome antigen 1	0.050	1.28
**20**	D3ZDR2	Chmp6 - Charged multivesicular body protein 6	0.030	1.27
**21**	Q5M884	Eci3 - enoyl-Coenzyme A delta isomerase	0.025	1.23
**22**	Q6P9U0	Serpinb6 - Serpin B6	0.040	1.20
**23**	D3ZQM0	Sf3a1 - Splicing factor 3A subunit 1	0.049	1.18
**24**	A0A0G2K7Y0	Cr1l - Complement component receptor 1-like protein	0.042	1.18
**25**	P35286	Rab13 - Ras-related protein Rab-13	0.040	1.17
**26**	P09895	Rpl5 - 60S ribosomal protein L5	0.040	1.17
**27**	B5DFN4	Pfdn5 - Prefoldin 5	0.038	1.12
**28**	P45479	Ppt1 - Palmitoyl-protein thioesterase 1	0.015	1.09
**29**	Q4KM69	Cops5 - COP9 (Constitutive photomorphogenic) homolog, subunit 5 (Arabidopsis thaliana)	0.034	0.93
**30**	Q66WT9	Picalm - Clathrin-assembly lymphoid myeloid leukemia protein	0.011	0.87
**31**	P11507	Atp2a2 - Sarcoplasmic/endoplasmic reticulum calcium ATPase 2 isoform b	0.036	0.87
**32**	P51635	Akr1a1 - Alcohol dehydrogenase [NADP(+)]	0.037	0.87
**33**	Q9R1T1	Banf1 - Barrier-to-autointegration factor	0.003	0.86
**34**	Q5VLR5	Erp44 - Endoplasmic reticulum resident protein 44 precursor	0.026	0.86
**35**	A0A0G2KAN7	Gls - Glutaminase kidney isoform, mitochondrial	0.049	0.86
**36**	P22509	Fbl - rRNA 2′-O-methyltransferase fibrillarin	0.031	0.86
**37**	A0A0G2KAW4	Thop1 - Thimet oligopeptidase	0.024	0.85
**38**	F2Z3Q8	Kpnb1 - Importin subunit beta-1	0.049	0.85
**39**	P19945	Rplp0 - 60S acidic ribosomal protein P0	0.027	0.84
**40**	D3ZUD8	Tm9sf3 - Protein Tm9sf3	0.009	0.84
**41**	Q9ESN0	Fam129a - Protein Niban	0.048	0.83
**42**	P62718	Rpl18a - 60S ribosomal protein L18a	0.041	0.83
**43**	Q4V882	Epn3 - Epsin-3	0.012	0.83
**44**	D3ZJH9	Me2 - Malic enzyme	0.019	0.83
**45**	D4A962	Hnrnpul1 - Heterogeneous nuclear ribonucleoprotein U-like protein 1	0.032	0.83
**46**	F7EWC1	Vasp - Vasodilator-stimulated phosphoprotein	0.034	0.82
**47**	F2W8B0	Comt - Catechol-O-methyltransferase	0.046	0.82
**48**	Q95571	RT1.A(u) - RT1.A(U) alpha chain	0.042	0.81
**49**	Q6AYF8	Serpinb9 - Serpin B9	0.031	0.81
**50**	Q9WU61	Clcc1 - Chloride channel CLIC-like protein 1 precursor	0.046	0.81
**51**	P51146	Rab4b - Ras-related protein Rab-4B	0.017	0.81
**52**	Q6P7R8	Hsd17b12 - Estradiol 17-beta-dehydrogenase 12	0.003	0.81
**53**	F1LPG9	Washc2c - WASH complex subunit 2C	0.014	0.79
**54**	Q5U2S7	Psmd3 - 26S proteasome non-ATPase regulatory subunit 3	0.039	0.79
**55**	A0A096MK73	Stmn1 - Stathmin	0.035	0.78
**56**	B1WC02	Ctps - CTP synthase 1	0.028	0.78

**57**	P17220	Psma2 - Proteasome subunit alpha type-2	0.048	0.78
**58**	Q63965	Sfxn1 - Sideroflexin-1	0.025	0.77
**59**	D3ZZR9	Fkbp2 - Peptidyl-prolyl cis-trans isomerase FKBP2 precursor	0.016	0.76
**60**	Q6P6G9	Hnrnpa1 - Heterogeneous nuclear ribonucleoprotein A1	0.009	0.76
**61**	M0RDJ4	Gmfb - Glia maturation factor beta	0.030	0.76
**62**	F1LT10	Afdn - Afadin	0.044	0.73
**63**	D4AB01	Hint2 - Histidine triad nucleotide binding protein 2	0.007	0.69
**64**	P04166	Cyb5b - Cytochrome b5 type B	0.020	0.68
**65**	A0A0G2JT00	Cuta - Divalent cation tolerant protein CUTA, isoform CRA_c	0.038	0.68
**66**	B2GVB7	Apeh - N-acylaminoacyl-peptide hydrolase	0.026	0.64
**67**	F1LQ09	Atl2 - ADP-ribosylation factor-like 6 interacting protein 2	0.004	0.64
**68**	A0A140TAJ3	Fubp1 - Far upstream element-binding protein 1	0.014	0.22
**69**	B1WBV1	Aida - Axin interactor, dorsalization-associated protein	0.010	0.21
**70**	Q4G079	Aimp1 - Aminoacyl tRNA synthase complex-interacting multifunctional protein 1	0.011	0.20
**71**	O54698	Slc29a1 - Equilibrative nucleoside transporter 1	0.005	0.15
**72**	P50503	St13 - Hsc70-interacting protein	0.040	0.07

Table 2. Comparative list of proteins in the BK-stimulated for 48h compared to controls.

Number	Accession	Name	q-value	fold change
**1**	F1LN30	Sumo4 - Small ubiquitin-related modifier	0.000	32.60
**2**	Q642E2	Rpl28 - 60S ribosomal protein L28	0.046	11.82
**3**	Q6DGF2	Clint1 - Clathrin interactor 1	0.002	10.22
**4**	Q499S2	Atp5g3 - ATP synthase F(0) complex subunit C3, mitochondrial	0.003	6.78
**5**	P60711	Actb - Actin	0.032	2.40
**6**	P61751	Arf4 - ADP-ribosylation factor 4	0.035	1.92
**7**	Q499N6	Ubxn1 - UBX domain-containing protein 1	0.003	1.90
**8**	A0A0G2K3P1	Transcription factor BTF3	0.023	1.78
**9**	Q2IBC6	Cav1 - Caveolin	0.047	1.74
**10**	F1LMD9	Gak - Cyclin-G-associated kinase	0.020	1.73
**11**	Q1JU69	Cops7a - ZH11 protein	0.001	1.70
**12**	D3ZAS1	ENSRNOG00000032289 - Protein RGD1562399	0.015	1.67
**13**	Q6PDU7	Atp5l - ATP synthase subunit g, mitochondrial	0.002	1.64
**14**	Q99MC0	Ppp1r14a - Protein phosphatase 1 regulatory subunit 14A	0.003	1.61
**15**	D4A8F2	Rsu1 - Ras suppressor protein 1	0.019	1.57
**16**	Q9QYW3	Mob4 - MOB-like protein phocein	0.035	1.50
**17**	P20070	Cyb5r3 - NADH-cytochrome b5 reductase 3	0.019	1.48
**18**	P70625	ZO-2 - Zonula occludens 2 protein	0.023	1.48
**19**	F1LNE5	Memo1 - Protein MEMO1	0.009	1.47
**20**	Q66HK3	Ptgs1 - Prostaglandin G/H synthase 1	0.035	1.45
**21**	Q6P7Q1	Bre - BRCA1-A complex subunit BRE	0.012	1.42
**22**	A0A0H2UHQ9	Synpo - Synaptopodin	0.038	1.41
**23**	Q5RKJ4	Fnta - Farnesyltransferase, CAAX box, alpha	0.045	1.39
**24**	A0A0G2JTX2	Praf2 - PRA1 family protein	0.016	1.38
**25**	O35264	Pafah1b2 - Platelet-activating factor acetylhydrolase IB subunit beta	0.031	1.37
**26**	B2GV92	Ptges3 - Ptges3 protein	0.049	1.37
**27**	P50545	Hck - Tyrosine-protein kinase HCK	0.039	1.36
**28**	A0A0G2KAI8	Drg2 - Developmentally-regulated GTP-binding protein 2	0.039	1.35
**29**	B0BN51	Snrpb - Small nuclear ribonucleoprotein-associated protein	0.015	1.34
**30**	D3Z8D7	Rps26 - Ribosomal protein S26	0.025	1.32
**31**	P61212	Arl1 - ADP-ribosylation factor-like protein 1	0.007	1.32
**32**	Q5XI19	Fermt2 - Fermitin family homolog 2	0.028	1.29
**33**	Q99PD6	Tgfb1i1 - Transforming growth factor beta-1-induced transcript 1 protein	0.022	1.28
**34**	I6L9G5	Rcn3 - Reticulocalbin 3	0.003	1.27
**35**	M0RD75	ENSRNOG00000049025 - 40S ribosomal protein S6	0.028	1.26
**36**	Q4V882	Epn3 - Epsin-3	0.014	1.25
**37**	A0A0G2K719	Ddx3x - DEAD-box helicase 3, X-linked	0.017	1.25
**38**	Q4AEF8	Copg1 - Coatomer subunit gamma-1	0.039	1.25
**39**	Q5EB77	Rab18 - Ras-related protein Rab-18	0.033	1.25
**40**	Q6P685	Eif2s2 - Eukaryotic translation initiation factor 2 subunit 2	0.035	1.24
**41**	Q5WQV5	Rdx - Radixin	0.011	1.23
**42**	Q6EV70	Pofut1 - GDP-fucose protein O-fucosyltransferase 1	0.028	1.21

**43**	G3V6P2	Dlst - Dihydrolipoamide S-succinyltransferase	0.004	1.20
**44**	Q6PDV7	Rpl10 - 60S ribosomal protein L10	0.031	1.19
**45**	D4A6W6	RGD1561333 - Protein RGD1561333	0.020	1.19
**46**	A0A0H2UHV4	Eif5b - Eukaryotic translation initiation factor 5B	0.022	1.19
**47**	D3Z941	Mars - methionine--tRNA ligase, cytoplasmic	0.022	1.18
**48**	F2Z3Q8	Kpnb1 - Importin subunit beta-1	0.035	1.17
**49**	Q5PPP1	Clta - Clathrin light chain	0.049	1.17
**50**	G3V8C4	Clic4 - Chloride intracellular channel protein	0.043	1.17
**51**	Q63584	Tmed10 - Transmembrane emp24 domain-containing protein 10	0.011	1.16
**52**	Q3ZB97	Ap2b1 - AP complex subunit beta	0.004	1.16
**53**	Q5RJR8	Lrrc59 - Leucine-rich repeat-containing protein 59	0.022	1.14
**54**	D3Z865	Mpdu1 - mannose-P-dolichol utilization defect 1 protein	0.021	1.13
**55**	Q4KM69	Cops5 - COP9 (Constitutive photomorphogenic) homolog, subunit 5	0.020	1.12
**56**	G3V6S3	Calu - Calumenin	0.010	1.11
**57**	Q66HF3	Etfdh - Electron transfer flavoprotein-ubiquinone oxidoreductase	0.047	1.10
**58**	Q5M7X1	Copb2 - Coatomer subunit beta′	0.032	1.10
**59**	G3V6P7	Myh9 - Myosin, heavy polypeptide 9, non-muscle	0.029	1.09
**60**	Q6LDS4	Sod1 - Superoxide dismutase [Cu-Zn]	0.034	0.91
**61**	A0A0G2JYL4	P4ha2 - Prolyl 4-hydroxylase subunit alpha 2	0.010	0.90
**62**	Q6P2A7	Flot1 - Flot1 protein	0.037	0.90
**63**	A7LNF8	RT1.A - MHC class I antigen	0.011	0.87
**64**	D3ZLH9	LOC680385 - Protein LOC680385	0.012	0.86
**65**	Q6P7A4	Psap - Prosaposin	0.047	0.85
**66**	Q64654	Cyp51 - Lanosterol 14-alpha demethylase	0.049	0.82
**67**	B1WBV1	Aida - Axin interactor	0.036	0.82
**68**	Q6AXM8	Pon2 - Serum paraoxonase/arylesterase 2	0.036	0.82
**69**	Q642A4	MGC94207 - UPF0598 protein C8orf82 homolog	0.039	0.82
**70**	Q95571	RT1.A(U) - RT1.A(U) alpha chain	0.005	0.81
**71**	A0A0A0MXU4	Emc2 - ER membrane protein complex subunit 2	0.011	0.81
**72**	Q9R1S6	Cd1d1 - CD1 antigen	0.045	0.80
**73**	B2RYG6	Otub1 - Ubiquitin thioesterase OTUB1	0.045	0.80
**74**	A0A0G2JSJ8	Fuca1 - Fucosidase, alpha-L-1, tissue, isoform CRA_a	0.021	0.80
**75**	Q7TPI8	Yipf5 - Protein YIPF5	0.042	0.80
**76**	B2RYQ5	Erh - Enhancer of rudimentary homolog	0.032	0.79
**77**	F1LR10	Lima1 - LIM domain and actin-binding protein 1	0.032	0.79
**78**	A1L1J8	Rab5b - Ras-related protein Rab-5B	0.007	0.78
**79**	Q566C5	Rassf4 - Ras association domain-containing protein 4	0.027	0.78
**80**	J7NUQ1	Irgm - Interferon-gamma-inducible GTPase Ifggd3 protein	0.019	0.77
**81**	P21571	Atp5j - ATP synthase-coupling factor 6, mitochondrial	0.040	0.77
**82**	M0R4V3	Snap23 - Synaptosomal-associated protein	0.037	0.76
**83**	G3V7L8	Atp6v1e1 - ATPase, H+ transporting, V1 subunit E isoform 1	0.030	0.76
**84**	Q4KM41	Ewsr1 - Ewing sarcoma breakpoint region 1	0.003	0.75
**85**	F7EWC1	Vasp - Vasodilator-stimulated phosphoprotein	0.020	0.75
**86**	P00388	Por - NADPH--cytochrome P450 reductase	0.044	0.75
**87**	Q4QQV4	Hars - histidyl-tRNA synthetase	0.023	0.74
**88**	A0A0G2K9T4	Macf1 - Microtubule-actin cross-linking factor 1	0.044	0.74
**89**	Q3KRE0	Atad3a - ATPase family AAA domain-containing protein 3	0.002	0.73
**90**	D4A7N1	Chchd6 - Coiled-coil-helix-coiled-coil-helix domain-containing protein 6	0.030	0.73
**91**	P00173	Cyb5a - Cytochrome b5	0.022	0.73
**92**	Q4KLJ1	Srsf7 - RCG61762, isoform CRA_a	0.021	0.73
**93**	Q68FY1	Nup35 - Nucleoporin NUP53	0.034	0.73
**94**	B2RYF6	Clptm1 - Cleft lip and palate associated transmembrane protein 1	0.005	0.72
**95**	Q5XIH3	Ndufv1 - NADH dehydrogenase	0.015	0.71
**96**	A0A0H2UHG0	Yars - Tyrosine--tRNA ligase	0.019	0.70
**97**	M0RBK1	Prps1l1 - Phosphoribosyl pyrophosphate synthetase 1-like 1	0.018	0.70
**98**	D3ZZ38	Snx18 - Sorting nexin-18	0.013	0.68
**99**	D4A8A0	Cad - CAD protein	0.037	0.68
**100**	P08009	Gstm3 - Glutathione S-transferase Yb-3	0.005	0.67
**101**	Q6AY21	G3bp2 - Ras GTPase-activating protein-binding protein 2	0.003	0.66
**102**	Q2YDU8	Spns1 - Protein spinster homolog 1	0.041	0.64
**103**	P07151	B2m - Beta-2-microglobulin	0.047	0.63
**104**	F2Z3S5	Rbpms - RNA-binding protein with multiple-splicing	0.035	0.62
**105**	P55770	Nhp2l1 - NHP2-like protein 1	0.002	0.61

**106**	B2LYI9	Tnc - Tenascin C	0.001	0.55
**107**	Q923Z2	Tpm1 - Tropomyosin 1, alpha, isoform CRA_a	0.000	0.54
**108**	P62078	Timm8b - Mitochondrial import inner membrane translocase subunit Tim8 B	0.007	0.49
**109**	P31643	Slc6a6 - Sodium- and chloride-dependent taurine transporter	0.022	0.48
**110**	D3ZCR3	Hmg1l1 - Protein RGD1560584	0.040	0.48
**111**	B2GVB7	Apeh - N-acylaminoacyl-peptide hydrolase	0.038	0.42
**112**	Q9Z1J7	Slc1a5 - Amino acid transporter	0.034	0.41
**113**	Q7TP42	Sec62 - Translocation protein SEC62	0.046	0.40
**114**	M0RBJ7	C3 - Complement C3	0.045	0.36
**115**	E9PU24	Dnah11 - Dynein-like protein 11	0.002	0.34
**116**	Q7TQ08	Scin - Scinderin	0.007	0.33
**117**	Q9WVK7	Hadh - Hydroxyacyl-coenzyme A dehydrogenase	0.020	0.26
**118**	Q99J82	Ilk - Integrin-linked protein kinase	0.003	0.25
**119**	D3ZCL3	Snrpc - U1 small nuclear ribonucleoprotein C	0.002	0.21
**120**	A0A140TAJ3	Fubp1 - Far upstream element-binding protein 1	0.047	0.20

Table 3. Comparative list of proteins in the leptin-stimulated for 24h compared to controls.

Number	Accession	Name	q-value	fold change
**1**	Q9QZA6	Cd151 - CD151 antigen	0.047	9.55
**2**	Q499S2	Atp5g3 - ATP synthase F(0) complex subunit C3, mitochondrial	0.001	7.92
**3**	B2RYL3	Tmem119 - Transmembrane protein 119 precursor	0.001	4.26
**4**	F1M6Q3	Col4a2 - Collagen type IV alpha 2 chain	0.027	3.53
**5**	Q566C5	Rassf4 - Ras association domain-containing protein 4	0.012	2.62
**6**	D4A4L5	Hbld1 - Uncharacterized protein LOC500694	0.015	2.61
**7**	F1LPS3	Nolc1 - Nucleolar and coiled-body phosphoprotein 1	0.005	2.42
**8**	A0A0H2UHV9	Copg2 - Coatomer subunit gamma	0.017	2.27
**9**	Q9JI92	Sdcbp - Syntenin-1	0.017	2.15
**10**	A0A0G2JSQ9	Pelp1 - Proline-, glutamic acid- and leucine-rich protein 1	0.023	2.15
**11**	Q6AY30	Sccpdh - Saccharopine dehydrogenase (putative)	0.022	1.96
**12**	D3ZSV1	Ift20 - Intraflagellar transport protein 20 homolog	0.010	1.94
**13**	Q5XIK2	Tmx2 - Thioredoxin-related transmembrane protein 2	0.021	1.93
**14**	Q5U2X6	Ccdc47 - Coiled-coil domain-containing protein 47	0.005	1.89
**15**	P47853	Bgn - Biglycan	0.019	1.86
**16**	D4AC36	Eif3f - Eukaryotic translation initiation factor 3 subunit F	0.037	1.74
**17**	M3ZCQ2	Snrnp200 - U5 small nuclear ribonucleoprotein 200 kDa helicase	0.005	1.69
**18**	P11505	Atp2b1 - Plasma membrane calcium-transporting ATPase 1	0.026	1.68
**19**	P50545	Hck - Tyrosine-protein kinase HCK	0.009	1.68
**20**	Q5RJK6	Inpp1 - Inositol polyphosphate 1-phosphatase	0.028	1.67
**21**	Q5RKH9	Derl1 - Derlin	0.024	1.67
**22**	F1LMD9	Gak - Cyclin-G-associated kinase	0.043	1.66
**23**	F1LM16	Serpine1 - Plasminogen activator inhibitor 1	0.029	1.65
**24**	A0A0G2JTL5	Pc - Pyruvate carboxylase, mitochondrial	0.012	1.58
**25**	P61751	Arf4 - ADP-ribosylation factor 4	0.048	1.57
**26**	F1LTJ5	Uncharacterized protein	0.029	1.57
**27**	P20070	Cyb5r3 - NADH-cytochrome b5 reductase 3	0.013	1.57
**28**	A0A097BVJ5	Cnp - 2′,3′-cyclic-nucleotide 3′-phosphodiesterase	0.010	1.57
**29**	A0A0G2K8T0	Asah1 - Acid ceramidase	0.008	1.56
**30**	B5DF91	Elavl1 - ELAV-like protein 1	0.025	1.51
**31**	D3ZQN7	Lamb1 - Laminin B1	0.046	1.47
**32**	A0A0H2UHQ0	Slc3a2 - 4F2 cell-surface antigen heavy chain	0.016	1.45
**33**	A0A0G2KAU7	Ltbp2 - Latent-transforming growth factor beta-binding protein 2	0.026	1.44
**34**	D3ZBL6	Nup160 - Nuclear pore complex protein Nup160	0.044	1.44
**35**	Q920A6	Scpep1 - Retinoid-inducible serine carboxypeptidase	0.014	1.44
**36**	P14604	Echs1 - Short-chain enoyl-CoA hydratase	0.004	1.43
**37**	P04762	Cat - Catalase	0.017	1.42
**38**	Q5XIB4	Ufsp2 - Ufm1-specific protease 2	0.039	1.42
**39**	D3ZIE9	Aldh18a1 - Delta-1-pyrroline-5-carboxylate synthase	0.014	1.37
**40**	Q66HK3	Ptgs1 - Prostaglandin G/H synthase 1	0.050	1.36
**41**	P08461	Dlat - Dihydrolipoyllysine-residue acetyltransferase component of pyruvate dehydrogenase complex	0.017	1.35
**42**	D3ZJ32	Esyt2 - Extended synaptotagmin-like protein 2	0.000	1.35
**43**	D3Z8D7	Rps26 - Ribosomal protein S26	0.001	1.35
**44**	B2GV15	Dbt - Lipoamide acyltransferase component of branched-chain alpha-keto acid dehydrogenase complex	0.038	1.34
**45**	Q32PW9	Psmc6 - Psmc6 protein	0.049	1.34
**46**	A0A0G2K7P7	Mtch2 - Mitochondrial carrier 2	0.025	1.33
**47**	Q63083	Nucb1 - Nucleobindin-1	0.046	1.33
**48**	Q6P7A9	Gaa - Lysosomal alpha-glucosidase precursor	0.000	1.32
**49**	P62634	Cnbp - Cellular nucleic acid-binding protein	0.034	1.32
**50**	Q4KM74	Sec22b - Vesicle-trafficking protein SEC22b	0.001	1.30
**51**	Q63965	Sfxn1 - Sideroflexin-1	0.003	1.29
**52**	B5DFG2	Hnrnpl - Hnrnpl protein	0.036	1.29
**53**	Q5RJR8	Lrrc59 - Leucine-rich repeat-containing protein 59	0.046	1.29
**54**	Q5EBA9	Suclg2 - Succinate-CoA ligase subunit beta	0.037	1.29
**55**	A0A0G2K251	Abcg3l1 - ATP-binding cassette, subfamily G (WHITE), member 3-like 1	0.010	1.28
**56**	P24368	Ppib - Peptidyl-prolyl cis-trans isomerase B	0.005	1.28
**57**	A0A0G2JSS9	Atl3 - Atlastin-3	0.039	1.28

**58**	D4A8M5	Snrpe - Small nuclear ribonucleoprotein E	0.001	1.27
**59**	D3ZQM0	Sf3a1 - Splicing factor 3A subunit 1	0.036	1.27
**60**	P97675	Enpp3 - Ectonucleotide pyrophosphatase/phosphodiesterase family member 3 Alkaline phosphodiesterase I Nucleotide pyrophosphatase	0.048	1.27
**61**	Q66HM2	Ap2a2 - AP-2 complex subunit alpha-2	0.001	1.27
**62**	Q6P7R8	Hsd17b12 - Estradiol 17-beta-dehydrogenase 12	0.017	1.27
**63**	M0R907	Snrpd3 - Small nuclear ribonucleoprotein D3	0.048	1.26
**64**	Q62991	Scfd1 - Sec1 family domain-containing protein 1	0.022	1.25
**65**	P04785	P4hb - Protein disulfide-isomerase	0.004	1.25
**66**	P06761	Hspa5 - 78 kDa glucose-regulated protein	0.000	1.25
**67**	G3V7T6	Sf3b1 - Splicing factor 3b, subunit 1	0.036	1.24
**68**	A0A0G2JXJ7	Esyt1 - Extended synaptotagmin-1	0.049	1.24
**69**	Q6AXR4	Hexb - Beta-hexosaminidase subunit beta	0.038	1.24
**70**	Q6P136	Hyou1 - Hyou1 protein	0.032	1.23
**71**	I6L9G6	Tardbp - TAR DNA binding protein	0.049	1.23
**72**	P52555	Erp29 - Endoplasmic reticulum resident protein 29	0.009	1.23
**73**	G3V8C3	Vim - Vimentin	0.048	1.22
**74**	Q9JJ54	Hnrpd - Heterogeneous nuclear ribonucleoprotein C	0.024	1.22
**75**	D3Z865	Mpdu1 - mannose-P-dolichol utilization defect 1 protein	0.043	1.21
**76**	A0A140TAI3	Hnrnpc - Heterogeneous nuclear ribonucleoprotein C	0.037	1.20
**77**	P70490	Mfge8 - Lactadherin	0.047	1.20
**78**	G3V6P2	Dlst - Dihydrolipoamide S-succinyltransferase	0.004	1.20
**79**	P35565	Canx - Calnexin	0.001	1.20
**80**	P97532	Mpst - 3-mercaptopyruvate sulfurtransferase	0.042	1.19
**81**	D3ZZR9	Fkbp2 - Peptidyl-prolyl cis-trans isomerase FKBP2 precursor	0.031	1.18
**82**	B2RYG2	Pck2 - Pck2 protein	0.035	1.18
**83**	G3V9W6	Aldh3a2 - Aldehyde dehydrogenase	0.028	1.17
**84**	O35821	Mybbp1a - Myb-binding protein 1A	0.021	1.17
**85**	A0A0A0MY09	Hsp90b1 - Endoplasmin	0.027	1.17
**86**	D3ZQ57	Plxnb2 - plexin-B2 precursor	0.019	1.17
**87**	G3V6H5	Slc25a11 - Mitochondrial 2-oxoglutarate/malate carrier protein	0.008	1.17
**88**	P61212	Arl1 - ADP-ribosylation factor-like protein 1	0.009	1.17
**89**	Q6PDV8	LOC100360057 - RCG31311	0.005	1.17
**90**	P18418	Calr - Calreticulin	0.030	1.17
**91**	A0A0G2K1L8	Basp1 - Brain acid soluble protein 1	0.046	1.16
**92**	Q63355	Myo1c - Unconventional myosin-Ic	0.000	1.16
**93**	Q6NYB7	Rab1A - Ras-related protein Rab-1A	0.042	1.16
**94**	B0BNG3	Lman2 - Vesicular integral-membrane protein VIP36 precursor	0.021	1.16
**95**	D3ZUY8	Ap2a1 - AP-2 complex subunit alpha-1	0.004	1.15
**96**	P56574	Idh2 - Isocitrate dehydrogenase [NADP]	0.033	1.15
**97**	A0A0G2JSZ5	Pdia6 - Protein disulfide-isomerase A6	0.040	1.14
**98**	F7F9U6	Plec - Plectin	0.007	1.13
**99**	Q6P2A5	Ak3 - GTP:AMP phosphotransferase AK3, mitochondrial	0.027	1.13
**100**	B0BN97	Txndc12 - Txndc12 protein	0.036	1.13
**101**	O08984	Lbr - Lamin-B receptor	0.039	1.13
**102**	Q5VLR5	Erp44 - Endoplasmic reticulum resident protein 44 precursor	0.040	1.12
**103**	Q5BJS6	Tmem50b - Transmembrane protein 50B	0.035	1.12
**104**	Q07936	Anxa2 - Annexin A2	0.010	1.11
**105**	Q9JLA3	Uggt1 - UDP-glucose:glycoprotein glucosyltransferase 1	0.010	1.10
**106**	G3V928	Lrp1 - Prolow-density lipoprotein receptor-related protein 1 precursor	0.023	1.09
**107**	B2RZ09	Pdia6 - Protein disulfide-isomerase A6	0.043	1.08
**108**	Q66HF3	Etfdh - Electron transfer flavoprotein-ubiquinone oxidoreductase	0.039	1.08
**109**	Q66WT9	Picalm - Clathrin-assembly lymphoid myeloid leukemia protein	0.008	0.91
**110**	F1LPK7	Pls3 - Plastin 3 (T-isoform), isoform CRA_a	0.049	0.90
**111**	Q4KM41	Ewsr1 - Ewing sarcoma breakpoint region 1	0.030	0.90
**112**	O55096	Dpp3 - Dipeptidyl peptidase 3	0.018	0.86
**113**	Q6IMZ3	Anxa6 - Annexin	0.048	0.85
**114**	G3V8B6	Psmd1 - 26S proteasome non-ATPase regulatory subunit 1	0.032	0.84
**115**	P24051	Rps27l - 40S ribosomal protein S27-like	0.032	0.83
**116**	P61972	Nutf2 - Nuclear transport factor 2	0.012	0.82
**117**	A0A096MK75	Rhog - Ras homolog family member G	0.015	0.82

**118**	C7C5T2	Pfkp - ATP-dependent 6-phosphofructokinase	0.045	0.82
**119**	P50399	Gdi2 - Rab GDP dissociation inhibitor beta	0.033	0.82
**120**	A0A0G2KAW4	Thop1 - Thimet oligopeptidase	0.020	0.81
**121**	Q5BKC3	Park7 - Park7 protein	0.024	0.81
**122**	Q08163	Cap1 - Adenylyl cyclase-associated protein 1	0.042	0.80
**123**	P27605	Hprt1 - Hypoxanthine-guanine phosphoribosyltransferase	0.006	0.80
**124**	Q62952	Dpysl3 - Dihydropyrimidinase-related protein 3	0.047	0.80
**125**	Q6PDW1	Rps12 - 40S ribosomal protein S12	0.009	0.79
**126**	B5DEN4	Ldha - L-lactate dehydrogenase	0.047	0.79
**127**	Q5M964	Fh - Fumarate hydratase 1	0.033	0.79
**128**	A0A0G2JZG7	Sars - Serine--tRNA ligase, cytoplasmic	0.041	0.79
**129**	A0A0G2JZ13	Cttn - Src substrate cortactin	0.032	0.78
**130**	E2RUH2	Rnh1 - Ribonuclease inhibitor	0.009	0.78
**131**	P45592	Cfl1 - Cofilin-1	0.009	0.78
**132**	D4A7U1	Zyx - Zyxin	0.009	0.77
**133**	P30835	Pfkl - 6-phosphofructokinase	0.010	0.77
**134**	A0A0G2K9A2	Arpc2 - Arp2/3 complex 34 kDa subunit	0.039	0.77
**135**	P34058	Hsp90ab1 - Heat shock protein HSP 90-beta	0.017	0.77
**136**	P23928	Cryab - Alpha-crystallin B chain	0.022	0.77
**137**	Q499Q4	Pgm1 - Phosphoglucomutase 1	0.020	0.76
**138**	Q6AYC4	Capg - Macrophage-capping protein	0.008	0.76
**139**	Q6P9U0	Serpinb6 - Serpin B6	0.043	0.76
**140**	Q5XIC6	Psmd12 - 26S proteasome non-ATPase regulatory subunit 12	0.010	0.76
**141**	E9PT82	Strn3 - Striatin-3	0.039	0.76
**142**	P48500	Tpi1 - Triosephosphate isomerase 1	0.038	0.75
**143**	D4AD15	Eif4g1 - Eukaryotic translation initiation factor 4 gamma, 1	0.036	0.75
**144**	P25113	Pgam1 - Phosphoglycerate mutase 1	0.022	0.75
**145**	B0BNA7	Eif3i - Eukaryotic translation initiation factor 3 subunit I	0.008	0.74
**146**	Q6P7S0	Pkm - Pyruvate kinase	0.029	0.74
**147**	Q99MI5	Srm - Spermidine synthase	0.045	0.74
**148**	Q66HF9	Lrrfip1 - Leucine-rich repeat flightless-interacting protein 1	0.028	0.74
**149**	D4A269	ENSRNOG00000005630 - Uncharacterized protein	0.042	0.74
**150**	Q6MG61	Clic1 - Chloride intracellular channel protein 1	0.010	0.74
**151**	G3V7X4	Abca2 - ATP-binding cassette sub-family A member 2	0.022	0.73
**152**	Q3T1J1	Eif5a - Eukaryotic translation initiation factor 5A-1	0.033	0.73
**153**	P13221	Got1 - Aspartate aminotransferase, cytoplasmic	0.017	0.72
**154**	P16617	Pgk1 - Phosphoglycerate kinase 1	0.044	0.72
**155**	A0A0H2UHG0	Yars - Tyrosine--tRNA ligase	0.002	0.71
**156**	A0A0G2K338	Fhl1 - Four and a half LIM domains protein 1	0.038	0.71
**157**	B2RYQ2	Ppp2r4 - Serine/threonine-protein phosphatase 2A activator	0.018	0.71
**158**	P47727	Cbr - Carbonyl reductase [NADPH] 1	0.024	0.71
**159**	P63004	Pafah1b1 - Platelet-activating factor acetylhydrolase IB subunit alpha	0.042	0.70
**160**	Q4QQV4	Hars - histidyl-tRNA synthetase, cytoplasmic	0.039	0.70
**161**	O35244	Prdx6 - Peroxiredoxin-6	0.024	0.70
**162**	P37397	Cnn3 - Calponin-3	0.022	0.70
**163**	R9PXV7	Ppp1r7 - Protein phosphatase 1 regulatory subunit 7	0.040	0.69
**164**	P47875	Csrp1 - Cysteine and glycine-rich protein 1	0.043	0.69
**165**	F1LPG9	Washc2c - WASH complex subunit 2C	0.031	0.69
**166**	D3ZF26	Tnks1bp1 - Protein Tnks1bp1	0.014	0.68
**167**	O70593	Sgta - Small glutamine-rich tetratricopeptide repeat-containing protein alpha	0.001	0.68
**168**	P62282	Rps11 - 40S ribosomal protein S11	0.036	0.68
**169**	A0A0G2JWK7	Tagln - Transgelin	0.037	0.68
**170**	O88656	Arpc1b - Actin-related protein 2/3 complex subunit 1B	0.006	0.67
**171**	F1M9V7	Npepps - Puromycin-sensitive aminopeptidase precursor	0.013	0.66
**172**	P05065	Aldoa - Fructose-bisphosphate aldolase A	0.034	0.66
**173**	Q6P4Z9	Cops8 - COP9 signalosome complex subunit 8	0.025	0.65
**174**	Q62766	kap12 - SSeCKS	0.033	0.64
**175**	D3ZBN3	Epha2 - Ephrin type-A receptor 2 precursor	0.023	0.64
**176**	B5DEH4	Uap1l1 - UDP-N-acetylhexosamine pyrophosphorylase-like protein 1	0.028	0.63
**177**	B2RYG6	Otub1 - Ubiquitin thioesterase OTUB1	0.001	0.63

**178**	P14669	Anxa3 - Annexin A3	0.012	0.63
**179**	Q66HR2	Mapre1 - Microtubule-associated protein RP/EB family member 1	0.005	0.61
**180**	F1LS72	Uba2 - SUMO-activating enzyme subunit 2	0.036	0.59
**181**	Q05982	Nme1 - Nucleoside diphosphate kinase A	0.036	0.54
**182**	Q923Z2	Tpm1 - Tropomyosin 1, alpha, isoform CRA_a	0.036	0.42
**183**	Q62658	Fkbp1a - Peptidyl-prolyl cis-trans isomerase FKBP1A	0.007	0.39
**184**	Q99J82	Ilk - Integrin-linked protein kinase	0.030	0.39
**185**	E9PU24	Dnah11 - Dynein-like protein 11	0.039	0.36
**186**	F7EZ89	Tbc1d15 - Protein Tbc1d15	0.010	0.34
**187**	D3ZCR3	Hmg1l1 - Protein RGD1560584	0.041	0.28
**188**	Q5XIF0	Tex264 - Testis-expressed sequence 264 protein	0.000	0.15
**189**	D3ZCI9	Myl10 - Myosin light chain 10	0.004	0.12

Table 4. Comparative list of proteins in the leptin-stimulated for 48h compared to controls.

Number	Accession	Name	q-value	fold change
**1**	D4A7C2	RGD1306349 - Formin 1	0.000	45.09
**2**	Q642E2	Rpl28 - 60S ribosomal protein L28	0.008	16.37
**3**	A0A0G2JUN7	Txnrd1 - Thioredoxin reductase 1, cytoplasmic	0.001	8.13
**4**	Q499S2	Atp5g3 - ATP synthase F(0) complex subunit C3	0.003	6.94
**5**	Q5XFW2	Trub2 - Probable tRNA pseudouridine synthase 2	0.018	5.81
**6**	D4AA43	Kbtbd5 - Kelch repeat and BTB domain-containing protein 5	0.016	5.62
**7**	O35112	Alcam - CD166 antigen	0.008	3.79
**8**	Q5I034	RGD1311899 - Uncharacterized protein C12orf43 homolog	0.010	2.74
**9**	F1LMD9	Gak - Cyclin-G-associated kinase	0.009	2.23
**10**	Q499N6	Ubxn1 - UBX domain-containing protein 1	0.005	2.15
**11**	D4AC36	Eif3f - Eukaryotic translation initiation factor 3 subunit F	0.005	2.05
**12**	B2GVB1	S100a6 - Protein S100	0.042	1.99
**13**	F1M6Q3	Col4a2 - Collagen type IV alpha 2 chain	0.016	1.97
**14**	P61751	Arf4 - ADP-ribosylation factor 4	0.019	1.97
**15**	D3ZAS1	ENSRNOG00000032289 - Protein RGD1562399	0.016	1.84
**16**	A0A0G2KAW7	Eif4h - Eukaryotic translation initiation factor 4H	0.038	1.63
**17**	Q99MC0	Ppp1r14a - Protein phosphatase 1 regulatory subunit 14A	0.041	1.56
**18**	D4A6C5	Arhgap1 - Rho GTPase activating protein 1	0.013	1.55
**19**	O35264	Pafah1b2 - Platelet-activating factor acetylhydrolase IB subunit beta	0.007	1.48
**20**	B2GV92	Ptges3 - Ptges3 protein	0.023	1.43
**21**	Q6P7Q1	Bre - BRCA1-A complex subunit BRE	0.045	1.43
**22**	F1LSW7	Rpl14 - 60S ribosomal protein L14	0.009	1.42
**23**	D4AE68	Gnaq - Guanine nucleotide binding protein, alpha q polypeptide	0.017	1.41
**24**	P50545	Hck - Tyrosine-protein kinase HCK	0.021	1.40
**25**	P47853	Bgn - Biglycan	0.039	1.38
**26**	Q6AYK8	Eif3d - Eukaryotic translation initiation factor 3 subunit D	0.005	1.37
**27**	Q3MHS9	Cct6a - T-complex protein 1 subunit zeta	0.005	1.36
**28**	F1LRI5	Gcn1l1 - GCN1 general control of amino-acid synthesis 1-like 1	0.006	1.36
**29**	A0A0G2JU77	Eif3k - Eukaryotic translation initiation factor 3 subunit K	0.040	1.34
**30**	Q6P792	Fhl1 - Four and a half LIM domains 1	0.017	1.34
**31**	B2RYN6	Ap1g1 - Adaptor-related protein complex 1, gamma 1 subunit, isoform CRA_b	0.023	1.33
**32**	B5DF91	Elavl1 - ELAV-like protein 1	0.031	1.33
**33**	P10111	Ppia - Peptidyl-prolyl cis-trans isomerase A	0.005	1.30
**34**	A0A140TAI8	Ahcyl1 - S-adenosylhomocysteine hydrolase-like protein 1	0.029	1.30
**35**	Q6P3V8	Eif4a1 - Eukaryotic initiation factor 4A-I	0.045	1.30
**36**	O35303	Dnm1l - Dynamin-1-like protein	0.017	1.30
**37**	Q3B8P5	Psmd8 - Psmd8 protein	0.007	1.29
**38**	E9PT66	Sf3b3 - Splicing factor 3B subunit 3	0.042	1.29
**39**	G3V6U3	Alg2 - Alpha-1,3-mannosyltransferase ALG2	0.049	1.28
**40**	D3Z8D7	Rps26 - Ribosomal protein S26	0.039	1.28
**41**	F1LNE5	Memo1 - Protein MEMO1	0.020	1.28
**42**	Q32PW9	Psmc6 - Psmc6 protein	0.006	1.27
**43**	P61212	Arl1 - ADP-ribosylation factor-like protein 1	0.002	1.27
**44**	D3ZRX9	Cnn2 - Calponin 2	0.034	1.27
**45**	Q6P685	Eif2s2 - Eukaryotic translation initiation factor 2 subunit 2	0.018	1.26
**46**	A0A0G2K9N3	Ddx3 - DEAD (Asp-Glu-Ala-Asp) box polypeptide 3	0.049	1.26
**47**	F1M6F4	RGD1564597 - Protein RGD1564597	0.019	1.26
**48**	P62634	Cnbp - Cellular nucleic acid-binding protein	0.031	1.25
**49**	A0A0G2JTN4	Pfas - Phosphoribosylformylglycinamidine synthase-like	0.039	1.25
**50**	A0A1B0GWY5	Arhgef2 - Rho guanine nucleotide exchange factor 2	0.021	1.25
**51**	G3V6H2	Prpf8 - pre-mRNA-processing-splicing factor 8	0.048	1.24
**52**	P56571	RGD1303003 - ES1 protein homolog	0.011	1.24
**53**	P19944	Rplp1 - 60S acidic ribosomal protein P1	0.014	1.22
**54**	F2Z3Q8	Kpnb1 - Importin subunit beta-1	0.015	1.22
**55**	Q4V882	Epn3 - Epsin-3	0.024	1.22
**56**	Q641Y8	Ddx1 - ATP-dependent RNA helicase DDX1	0.027	1.21
**57**	Q9JMB5	Adrm1 - Proteasomal ubiquitin receptor ADRM1	0.028	1.21
**58**	P09456	Prkar1a - cAMP-dependent protein kinase type I-alpha regulatory subunit	0.022	1.21
**59**	D4A9Q3	RGD1563570 - Protein RGD1563570	0.007	1.20
**60**	G3V826	Tkt - Transketolase	0.035	1.20

**61**	Q4AEF8	Copg1 - Coatomer subunit gamma-1	0.048	1.20
**62**	Q5RJR8	Lrrc59 - Leucine-rich repeat-containing protein 59	0.012	1.20
**63**	A0A0G2JV05	Mroh1 - Maestro heat-like repeat family member 1	0.019	1.19
**64**	F1LN59	Eif4g2 - Eukaryotic translation initiation factor 4 gamma, 2	0.010	1.19
**65**	A0A0G2K719	Ddx3x - DEAD-box helicase 3, X-linked	0.036	1.18
**66**	Q63413	Ddx39b - Spliceosome RNA helicase Ddx39b	0.039	1.18
**67**	Q9ESN0	Fam129a - Protein Niban	0.005	1.17
**68**	Q641Z6	Ehd1 - EH domain-containing protein 1	0.039	1.16
**69**	D3ZBL6	Nup160 - Nuclear pore complex protein Nup160	0.014	1.16
**70**	B0K031	Rpl7 - 60S ribosomal protein L7	0.036	1.16
**71**	F1LRA1	Lman1 - Protein ERGIC-53	0.019	1.16
**72**	A0A0G2K0Z7	Gpd2 - Glycerol-3-phosphate dehydrogenase, mitochondrial	0.011	1.15
**73**	Q5PPP1	Clta - Clathrin light chain	0.014	1.14
**74**	C0JPT7	Flna - filamin-A	0.015	1.14
**75**	G3V8C4	Clic4 - Chloride intracellular channel protein	0.041	1.13
**76**	P62161	Calm1 - Calmodulin	0.005	1.11
**77**	Q07936	Anxa2 - Annexin A2	0.039	1.07
**78**	P06761	Hspa5 - 78 kDa glucose-regulated protein	0.001	1.06
**79**	O88656	Arpc1b - Actin-related protein 2/3 complex subunit 1B	0.041	0.96
**80**	Q66HF9	Lrrfip1 - Leucine-rich repeat flightless-interacting protein 1	0.047	0.93
**81**	P11884	Aldh2 - Aldehyde dehydrogenase, mitochondrial precursor	0.013	0.92
**82**	P15999	Atp5a1 - ATP synthase subunit alpha, mitochondrial precursor	0.035	0.92
**83**	A0A0G2K7K2	Aifm1 - Apoptosis-inducing factor 1, mitochondrial	0.019	0.90
**84**	D3ZJ32	Esyt2 - Extended synaptotagmin-like protein 2	0.032	0.89
**85**	P81155	Vdac2 - Voltage-dependent anion-selective channel protein 2	0.010	0.89
**86**	P08009	Gstm7 - Glutathione S-transferase Yb-3	0.050	0.87
**87**	A0A0G2JYC7	Hdgfrp2 - Uncharacterized protein	0.009	0.84
**88**	P29266	Hibadh - 3-hydroxyisobutyrate dehydrogenase	0.024	0.83
**89**	Q6P6W1	Lamp2 - Lysosome-associated membrane glycoprotein 2	0.022	0.83
**90**	F1LUV9	Ncam1 - Neural cell adhesion molecule 1	0.037	0.82
**91**	P11507	Atp2a2 - Sarcoplasmic/endoplasmic reticulum calcium ATPase 2 isoform b	0.026	0.81
**92**	D3ZTX0	Tmed7 - Transmembrane emp24 domain-containing protein 7	0.049	0.81
**93**	Q6AY23	Pycr2 - Pyrroline-5-carboxylate reductase 2	0.004	0.81
**94**	Q566C5	Rassf4 - Ras association domain-containing protein 4	0.023	0.80
**95**	D3ZZM8	LOC680432 - Uncharacterized protein	0.027	0.80
**96**	Q68FZ8	Pccb - Propionyl coenzyme A carboxylase, beta polypeptide	0.004	0.80
**97**	A0A0G2JSJ8	Fuca1 - Fucosidase, alpha-L-1, tissue, isoform CRA_a	0.017	0.79
**98**	Q5XIU9	Pgrmc2 - Membrane-associated progesterone receptor component 2	0.020	0.78
**99**	Q95571	RT1.A(u) - RT1.A(U) alpha chain	0.031	0.78
**100**	Q498C8	Rer1 - Protein RER1	0.034	0.77
**101**	D3ZSL2	Abracl - Uncharacterized protein LOC685045	0.003	0.76
**102**	A0A140TAI9	Leprotl1 - Leptin receptor overlapping transcript-like 1	0.039	0.76
**103**	Q3KRE0	Atad3a - ATPase family AAA domain-containing protein 3	0.033	0.76
**104**	D4A7D7	H6pd - GDH/6PGL endoplasmic bifunctional protein precursor	0.036	0.75
**105**	Q6P6G9	Hnrnpa1 - Heterogeneous nuclear ribonucleoprotein A1	0.034	0.75
**106**	B2RYF6	Clptm1 - Cleft lip and palate associated transmembrane protein 1	0.028	0.74
**107**	A7LNF8	RT1.A - MHC class I antigen	0.034	0.73
**108**	P32551	Uqcrc2 - Cytochrome b-c1 complex subunit 2, mitochondrial	0.037	0.73
**109**	D4AC70	Col8a1 - Collagen alpha-1(VIII) chain precursor	0.009	0.73
**110**	Q6P6Q5	App - Amyloid beta A4 protein	0.042	0.71
**111**	Q5M7V8	Thrap3 - Thyroid hormone receptor-associated protein 3	0.002	0.68
**112**	F1M866	Sorbs1 - Sorbin and SH3 domain-containing protein 1	0.014	0.68
**113**	Q63524	Tmed2 - Transmembrane emp24 domain-containing protein 2	0.039	0.65
**114**	P55770	Nhp2l1 - NHP2-like protein 1	0.033	0.65
**115**	D3ZXJ5	Eftud1 - Elongation factor Tu GTP-binding domain-containing protein 1	0.006	0.65
**116**	D4A962	Hnrnpul1 - Heterogeneous nuclear ribonucleoprotein U-like protein 1	0.012	0.63
**117**	P62078	Timm8b - Mitochondrial import inner membrane translocase subunit Tim8 B	0.001	0.63
**118**	Q923Z2	Tpm1 - Tropomyosin 1, alpha, isoform CRA_a	0.000	0.56
**119**	B2LYI9	Tnc - Tenascin C	0.003	0.54
**120**	D3ZFQ8	Cyc1 - Cytochrome c-1	0.016	0.49
**121**	Q5RJY4	Dhrs7b - Dehydrogenase/reductase SDR family member 7B	0.011	0.43
**122**	A0A0G2K103	Sptlc2 - Serine palmitoyltransferase, long chain base subunit 2	0.020	0.33
**123**	G3V9M6	Fbn1 - Fibrillin-1 precursor	0.035	0.31

**124**	P63012	Rab3a - Ras-related protein Rab-3A	0.001	0.27
**125**	G3V6S2	Aco1 - Aconitate hydratase	0.030	0.27
**126**	A0A140TAJ3	Fubp1 - Far upstream element-binding protein 1	0.050	0.24
**127**	P08082	Cltb - Clathrin light chain B	0.000	0.08

Supplementary methods:

Extraction and tryptic digestion of proteins

VSMC from control, BK 10^{-7} M (24h, or 48h), and Leptin 3.1 nM (24h, or 48h) were homogenized using beads beater (Beadbug microtube homogenizer, Benchmark Scientific, Edison, NJ) in 5% sodium deoxycholate (SDC) solution, followed by sonication on ice for 30 min and centrifugation at 21,000 g for 10 min. The resulting supernatant was diluted ten times with 50 mM ammonium bicarbonate (ABC) buffer, and total protein concentration was determined by BCA protein assay kit (Thermo Scientific/Pierce, Rockford, IL) according to the user's manual.

Aliquots of 10 μg extracted proteins from each sample were denatured at 80 oC for 10 min followed by adding 200 mM dithiothreitol (DTT) at a ratio of 1:40 (DTT: sample, v/v) and incubation at 60oC for 45 min. The resulting reduced proteins were then alkylated by adding iodoacetamide (IAA, 200 mM solution) at a ratio of 1:10 (IAA: sample, v/v) and incubation at 37°C in the dark for 45 min. Additional DTT quenched excessive IAA by incubating the solution at 37°C for 30 min. Trypsin (Promega, Madison, WI) was added at a ratio of 1:25 (enzyme: proteins, w/w) into samples and incubated at 37°C for 18 h followed by addition of formic acid at a final concentration of 0.5% to quench the enzymatic reaction and precipitate SDC at the same time. The supernatant containing tryptic digests were collected by centrifugation at 21,000 g for 10 min. Tryptic digested peptides were dried and then resuspended in an aqueous solution containing 2% acetonitrile (ACN) and 0.1% formic acid (FA) for Liquid chromatography-electrospray ionization-tandem mass spectrometry (LC-ESI-MS/MS) analysis.

Liquid Chromatography-Mass Spectroscopy/Mass Spectroscopy analysis

Aliquots (1μg) of tryptic digested samples were subjected to LC-ESI-MS/MS analysis. The analysis was performed using a Dionex Ultimate 3000 nano-LC system (Thermo Scientific, San Jose, CA) interfaced to an LTQ Orbitrap Velos mass spectrometer (Thermo Scientific, San Jose, CA) equipped with a nano-ESI source. Injected peptides were first purified on-line at a flow rate of 3 μl/min using a C18 Acclaim PepMap 100 trap column (75 μm I.D. × 2 cm, 3 μm particle sizes, 100 Å pore sizes, Thermo Scientific, San Jose, CA). Peptides were then separated on a C18 Acclaim PepMap RSLC column (75 μm I.D. × 15 cm, 2 μm particle sizes, 100 Å pore sizes, Thermo Scientific, San Jose, CA). The column temperature was maintained at 29.5 °C, and the flow rate was 350 nl/min during the separation. The mobile phase consisted of solution A (97.9% water/2% ACN/0.1% FA) and solution B (99.9% ACN/0.1% FA). The separation of peptides was achieved by following gradient of solution B: 5% over 10 min, 5–20% over 55 min, 20–30% over 25 min, 30–50% over 20 min, 50–80% over 1 min, 80% over 4 min, 80–5% over 1 min and 5% over 4 min. Data-dependent acquisition mode with two scan events was employed for MS/MS analysis. The first scan event was a full MS scan of 400-2000 *m/z* at a mass resolution of 15,000. In the second scan event, ten most intense ions detected in the first scan event were selected with an isolation width of 3.0 *m/z* to perform CID MS/MS. The normalized collision energy (CE) was set to 35%, and an activation Q value was 0.250. The dynamic exclusion was set to have a repeat count of 2, repeat duration of the 30s, exclusion list size 200 and exclusion duration of 90s.

Immunocytochemistry of RASMC:

Immunocytochemistry staining was utilized to assess levels of Leptin Receptor (LeptR) expression in the different BK time-point stimulations (24h and 48h). Briefly, RASMC were permeablized with 0.2% Triton-X100 for 30 min and blocked with 0.5% BSA for 30 min. Cells were stained with antibodies against Leptin receptor (ab5593, Abcam) antibody for overnight incubation

in cold room, then washed 4 times with PBS for 5 min each and then incubated with anti-rabbit conjugated to FITC (ab6717, Abcam) for 1 h at room temperature, then washed 4 times with PBS for 5 min each. Nuclei of the cells were stained with Bisbenzimide (Hoechst 33342 stain, Sigma-Aldrich). Images of tissues were acquired using Laser Scanning Confocal Microscope (Leica Microsystems, Cambridge, UK).
